# Three-dimensional gravity modelling of a Quaternary overdeepening fill in the Bern area of Switzerland discloses two stages of glacial carving

**DOI:** 10.1038/s41598-022-04830-x

**Published:** 2022-01-27

**Authors:** D. Bandou, F. Schlunegger, E. Kissling, U. Marti, M. Schwenk, P. Schläfli, G. Douillet, D. Mair

**Affiliations:** 1 Institute of Geological Sciences, University of Bern, Bern, Switzerland; 2 Department of Earth Sciences, ETH Zürich, Zurich, Switzerland; 3 Landesgeologie swisstopo, Bern, Switzerland; 4 Institute of Plant Sciences, University of Bern, Bern, Switzerland

**Keywords:** Geology, Geomorphology, Geophysics, Sedimentology

## Abstract

The geometry of glacial overdeepenings on the Swiss Plateau close to Bern was inferred through a combination of gravity data with a 3D gravity modelling software. The target overdeepenings have depths between 155 and > 270 m and widths between 860 and 2400 m. The models show incisions characterized by U-shaped cross-sectional geometries and steep to over-steepened lateral flanks. Existing stratigraphic data reveals that the overdeepenings were formed and then filled during at least two glacial stages, which occurred during the Last Glacial Maximum (LGM) within the Marine Isotope Stage (MIS) 2, and possibly MIS 6 or before. The U-shaped cross-sectional geometries point towards glacial erosion as the main driver for the shaping of the overdeepenings. The combination of the geometries with stratigraphic data suggests that the MIS 6 (or older) glaciers deeply carved the bedrock, whereas the LGM ice sheet only widened the existing valleys but did not further deepen them. We relate this pattern to the different ice thicknesses, where a thicker MIS 6 ice was likely more powerful for wearing down the bedrock than a thinner LGM glacier. Gravity data in combination with forward modelling thus offers robust information on the development of a landscape formed through glaciers.

## Introduction

**Figure 1:**
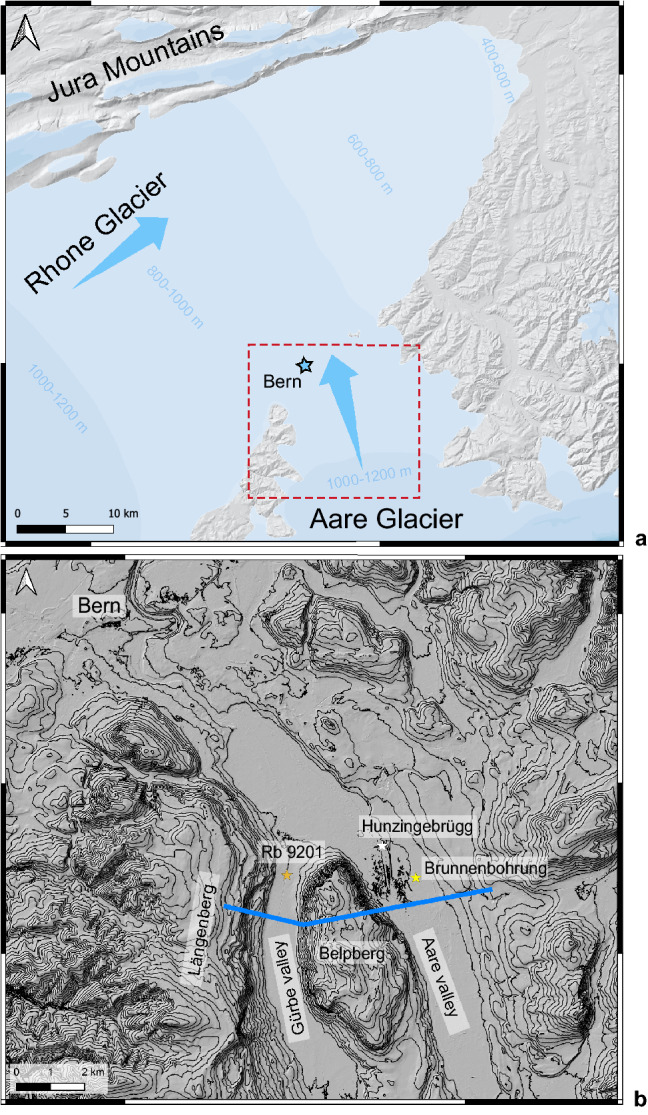
LGM glaciers and modern topography. (**a**) Map^21^ showing the extent and thicknesses of the glaciers that shaped the landscape of the study area during LGM times. This map was taken and modified from the openly accessible database of swisstopo (© swisstopo). (**b**) Topographic map illustrated as hill shaded DEM (SwissAlti3D, © swisstopo), showing the drilling locations in the Gürbe valley (RB9201) and two drillings in the Aare valley (Brunnenbohrung Münsingen, drilling Hunzigebrügg). The equidistance of the contour lines is 20 m. The map also shows the bedrock ridges (Belpberg, Längenberg) that framed the flow of the glaciers. Both datasets are available from the openly accessible database of swisstopo.

Overdeepenings are bedrock depressions with their deepest part below the base level of today’s rivers^1–4^. Overdeepenings occur in formerly glaciated plateaus and mountain belts around the world^1–4^. They have been inferred for the time as far back as the Ordovician^5,6^, and for the major valleys and the foreland surrounding the European Alps^7–9^ including the Central Swiss Alps^3,7,10–14^. There, overdeepenings are filled with Quaternary sediments and/or host modern lakes^8^. They may reach depths of several hundred meters (e.g., in the Rhone valley^3,15^), and cross-sectional widths of a few kilometres. Most of the overdeepenings feature downstream-directed adverse slopes along their thalwegs. Because the sedimentary fill inhibits any direct observations of their geometry, interpretations about their formation have been subject to controversial debates in the literature^16^. Indeed, overdeepenings may result from one or a combination of various erosional mechanisms such as glacial carving^4,8,16,17^, erosion by pressurized meltwater^11,17^, or a combination of both^16,17^. However, as noted before^17^, the main key to unravel the erosional mechanisms leading to the carving of overdeepenings is offered by the shape and morphometry of the bedrock such as e.g., V- or U-shaped cross-sectional geometries. In the past decades, multiple geophysical methods including seismic, geoelectric, and gravimetric surveys have been employed to disclose such geometries underneath overdeepening fills^18–20^. These surveys have been complemented with the results of shallow drillings, which yielded a complex picture of these overdeepenings^12^, yet with a relatively low resolution particularly of their lateral flanks and their bases. This is also the case for the Bern region, situated in the Swiss Plateau on the northern margin of the Alps (Fig. 1). This area was in the confluence area of two large glaciers (Aare and Rhone glaciers, Fig. 1a)^21^ during the past glacial periods, which resulted in the formation of multiple overdeepenings. South of Bern, two troughs that are > 150 m-deep and filled by Quaternary sediments underlay the 1 km-wide Gürbe valley in the West and the 2.5 km-wide Aare valley in the East^12–14^. Both valleys are flat and roughly S–N orientated (Fig. 1b). They are separated by an approximately 2.9 km-wide and > 350 m-high bedrock ridge referred to as the Belpberg, which has the same orientation as the valleys. Such geometric constraints offer ideal conditions for inferring the details of the overdeepenings’ geometries using gravity data^18^, which is accomplished in this study.

Here, we conducted a gravity survey for the area south of Bern (Fig. 1) to reconstruct the cross-sectional geometric details of the lateral flanks and the bottom of the target overdeepenings. We used the gravity data to calculate the Bouguer anomalies caused by the overdeepening fills. We additionally developed and applied a forward modelling program, named Prisma, to conduct this reconstruction. We then compiled and re-interpreted stratigraphic data of the overdeepening fills to propose a scenario of how the overdeepenings were formed within the framework of Alpine glaciations.

## Results

### Landscape morphometry and geology

The Belpberg mountain, situated in the centre of our gravity survey, has a trapezoidal cross-sectional geometry with c. 15° steep flanks on both sides and a rather flat top at 893 m a.s.l. The mountain flank bordering the Gürbe valley on its western side (Längenberg) is steep (Fig. 1b), exposes the bedrock in some places and hosts landslide deposits, while the eastern margin of our survey, facing the Aare valley, is smooth and characterized by hills several hundreds of meters wide. The valley floors themselves are flat and situated at an elevation of c. 520 m a.s.l.

**Figure 2:**
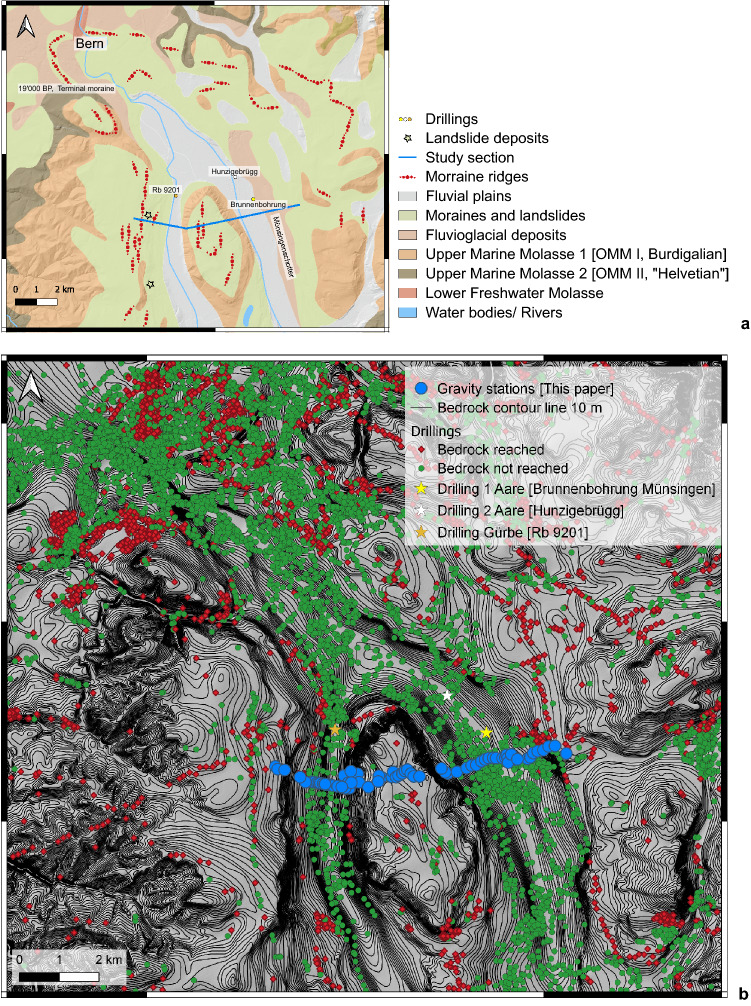
Geology of the region. (**a**) Geological map^22,25^, showing LGM moraines, pre-LGM gravel (Münsingenschotter), landslides deposits on the western flank of the Gürbe valley, the bedrock lithology, and the Holocene deposits covering the Aare and Gürbe valley floors. The bedrock overlying the Aare and Gürbe overdeepenings comprise a monotonous sequence of Burdigalian Upper Marine Molasse sandstones^22,25^. This map was obtained and modified from the openly accessible database of swisstopo (© swisstopo). (**b**) Map showing the contour lines (equidistance of 10 m) of the bedrock underneath the Quaternary cover. This map was reproduced using the openly accessible digital dataset for the bedrock topography of the canton Bern (https://www.geo.apps.be.ch/de/; Karten; Felsrelief), and it is also available from the openly accessible database of swisstopo (© swisstopo). This dataset was originally produced by Reber and Schlunegger^12^. Some drillings did reach the bedrock (red dots) and thus offer good constraints on the bedrock topography. Most of the drillings, however, did not reach the bedrock and yield minimum constraints on the depths of the overdeepening contour lines (green dots). The map has an uncertainty between 20 and 50 m. The blue dots illustrate the location of the stations that are used in this study. This map is also available from the openly accessible database of swisstopo (© swisstopo).

Mapping showed that the Belpberg comprises a suite of Burdigalian Upper Marine Molasse (UMM) sandstones with conglomerate interbeds^22^ (Fig. 2a). Mapping also revealed that after the Last Glacial Maximum (LGM) the Aare glacier deposited moraines on the top and the flank of the Längenberg (situated on the western margin of the Gürbe valley) and on the Belpberg. Additionally, upon retreating from its maximum extent, the Aare glacier formed a terminal moraine in the city of Bern at c. 545 m a.s.l., which was dated with cosmogenic ^10^Be to 19,000 years before present (BP)^23^. The Aare glacier then retreated towards the Alps, and the study area was ice-free by 17,000 years BP at the latest^24^. The eastern border of the Aare valley also exposes a Quaternary suite of gravel and mud, which has been referred to as the Münsingenschotter^25^ or Uttigen-Bümberg-Schotter, and which predate the LGM^26^.

Overdeepenings have been encountered through drillings beneath the Aare and the Gürbe valleys (Fig. 2b). They have steep flanks and possibly flat bases, with predicted depths at elevations of 380 ± 25 m and 260 ± 25 m a.s.l. beneath the Gürbe and Aare valleys, respectively^12–14^. Yet, the details on the geometry are poorly constrained due to a lack of drillings and seismic surveys^12^.

### Gravity profiling and estimation of the bedrock density

**Figure 3:**
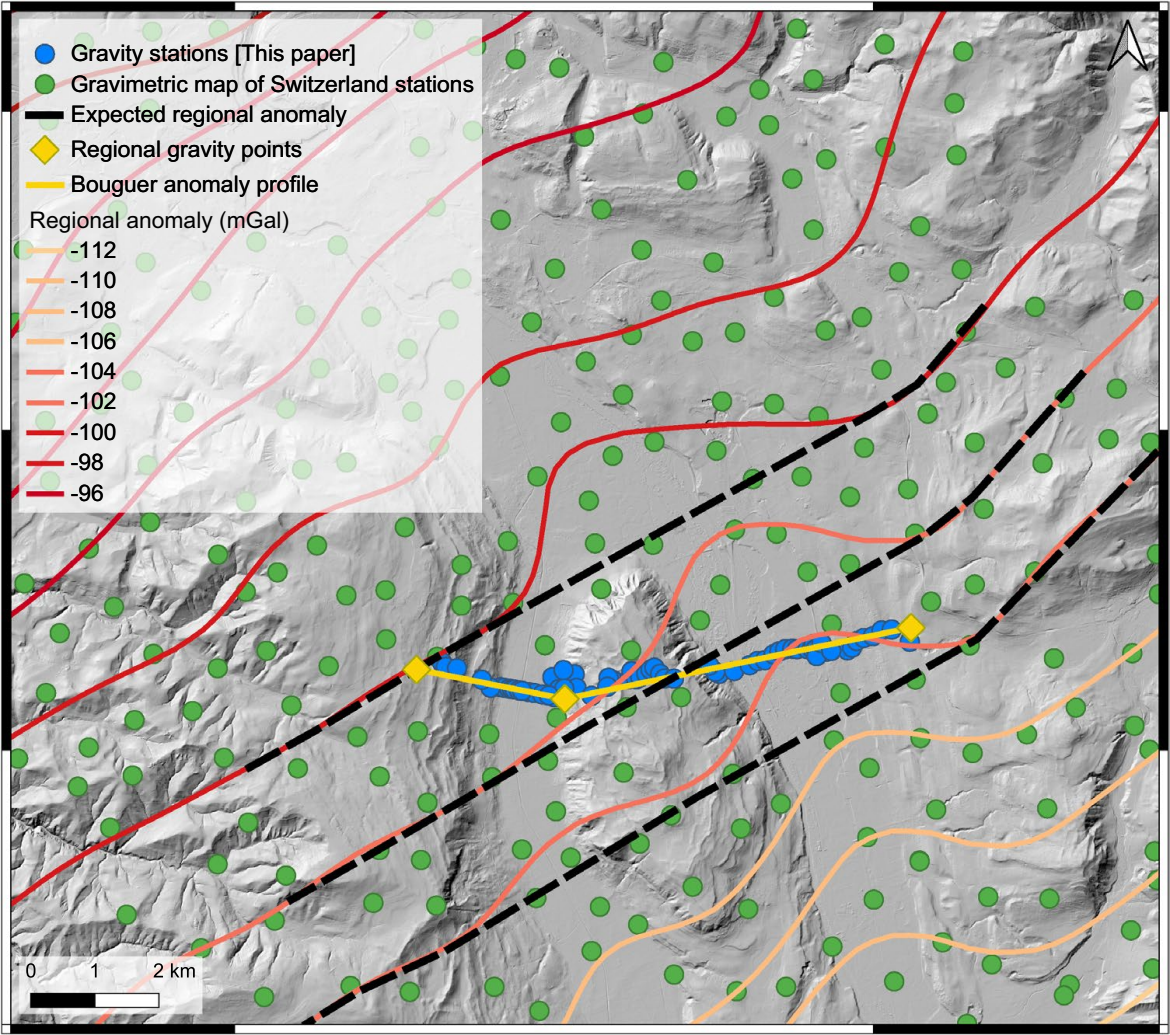
Gravity data. Regional gravity map, Bouguer anomaly contour lines and location of our stations. The map and the contour lines are from the openly accessible database of swisstopo (‘Carte gravimétrique de la Suisse (Anomalies de Bouguer) 1: 500,000, © swisstopo)^27^. The dashed black line indicates the estimated regional trend where the local anomalies related to the overdeepening fills have been removed. The yellow line shows the location of our section. The yellow diamonds indicate locations where our section starts or where it changes the direction. At these sites, we extracted the values of the regional gravity trend along the dashed black line, which is illustrated in Fig. 4a.

**Figure 4:**
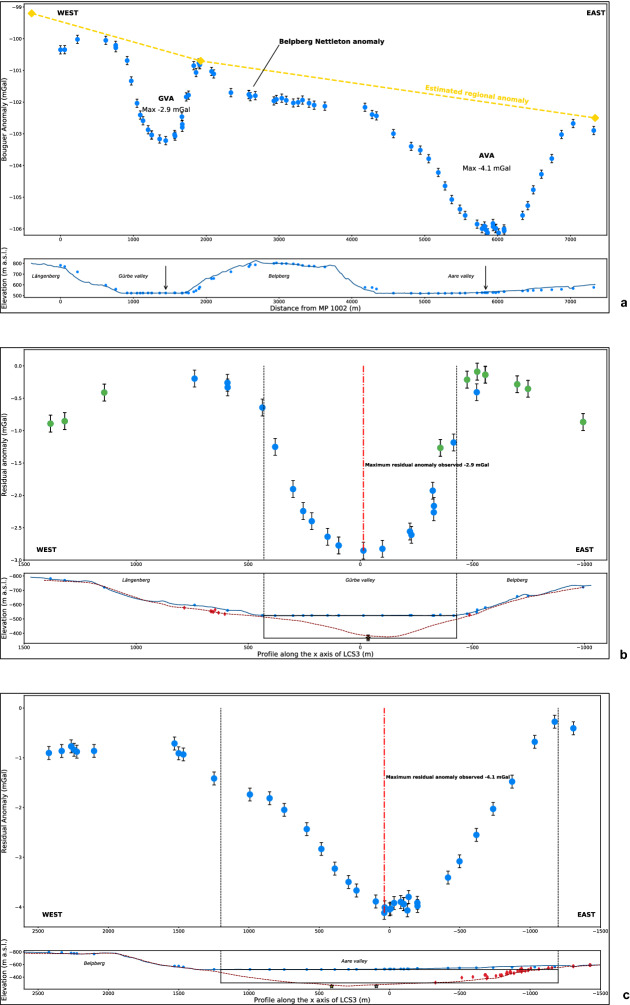
Bouguer and residual anomalies. (**a**) Top: Regional Bouguer anomaly plot. The blue dots indicate the Bouguer anomaly measured at a station; the black bar is the related uncertainty of +/− 0.13 mGal. The yellow diamonds indicate the points used to estimate the regional Bouguer gravity (dashed yellow line, see Fig. 3), which would be measured if there were no local anomalies. These anomalies are the Gürbe Valley Anomaly (GVA) of − 2.9 mGal, the Aare Valley Anomaly (AVA) of − 4.1 mGal, and the Belpberg Nettleton anomaly (Tables S2 and S3). The western end of the profile points to an anomaly possibly due to the bedrock underlying the Längenberg and the moraine cover. This anomaly is not further used. Bottom: Plot of the elevation of the gravity profile (see Fig. 3 for location) with the stations’ elevations, represented by the blue line and dots, respectively. Due to the projection of the elevation profile onto a line some stations are offset. The black arrows indicate the stations with the maximum GVA and AVA values. (**b**) Top: Residual anomaly plot of the Gürbe valley. The values are the difference between the measured Bouguer anomalies and the regional trend. The blue dots represent the stations < 100 m away from the gravity profile, whereas the green dots are the stations at > 100 m distance. The black dashed lines indicate the border of the valley floor, which delimit our models’ widths. Bottom: The bedrock profile of Reber and Schlunegger^12^ is indicated by the red dashed line with red diamonds showing the drillings that reached the bedrock. These are used as constraints for the modelling. The black rectangle is the cross-section of the first model approximating the geometry of the valley. (**c**) Top: Residual anomaly plot for the Aare valley, calculated in the same way as for the Gürbe valley. Bottom: Elevation and bedrock model profiles together with cross section of the prism that is used as a first order model of the Aare valley (see (b) for the legend). Note that the stations are not differentiated by distance to the profile because this will not yield an added value. Yellow stars in (b) and (c) indicate the depths of drillings shown in Figs. 2 and 6.

The compilation of existing gravity data from the Gravimetric map of Switzerland (1:500,000)^27^ clearly illustrate the gravity effects related to the sedimentary fills of the overdeepened valleys (Fig. 3). In particular, the regional isolines, which are oriented SE-NW and thus parallel to the strike of the Swiss Alps, are locally distorted around the Aare and Gürbe valleys and reflect gravity value deflections by a few mGal compared to the adjacent margins. This pattern is attributed to the relatively lower density of the Quaternary overdeepening fill beneath the two valleys^18,28^. These isolines, however, are based on measurements at stations that have a spacing between them that is too large to precisely identify the local gravity effects of the overdeepening fill. For this survey, additional data collected at 78 sites, along a section that is oriented perpendicular to the orientation of the overdeepenings and that also includes the Belpberg, is used to refine this pattern (Figs. 2b, 3 and 4a, Fig. S1, Table S1). For each valley, we then determined the residual anomaly caused by the overdeepening fill, which is the difference between the expected regional gravity and the observed regional anomaly (Fig. 4a). The results (Tables S2 and S3) show two distinct residual anomalies, one above the Gürbe valley with a maximum of − 2.9 mGal (Fig. 4b) and another one above the Aare valley with a maximum of − 4.1 mGal (Fig. 4c). Replicate measurements at 11 sites yielded an uncertainty of ± 0.13 mGal for this entire survey (Table S1).

The residual anomaly over the Belpberg additionally demonstrates that the bedrock density is slightly lower than the standard density of 2670 kg/m^3^. The application of Nettleton’s method^29^ (see also methods section) documents that a rock density of 2500 kg/m^3^ best eliminates the anomaly caused by the Belpberg topography (Fig. S2; Table S2). Therefore, we used this density of the UMM bedrock to model the residual Bouguer anomaly across the Gürbe and Aare valleys (see following sections).

### Estimation of the density contrast between the Molasse bedrock and the overdeepening fill

We estimated the density contrast between the overdeepening fill and the Molasse bedrock through representing the overdeepening fill by a single prism^18^, which parallels the orientation of the corresponding valley (Fig. S4a). The results show that a thickness of c. 155 m for the Quaternary suite in combination with a density contrast of 500 kg/m^3^ best fits the maximum residual anomaly of − 2.9 mGal in the Gürbe valley (Fig. S4b). For the Aare valley, the situation is more complicated because the residual anomalies are slightly asymmetric (Fig. 4c), and the bedrock model^12^ indicates that the bedrock topography contour lines are curved over short distances of < 100 m (Fig. S4a). Therefore, we used the results of the much simpler Gürbe valley and employed a density contrast of 500 kg/m^3^, which yields a minimum thickness of 210 m for the fill of the Aare overdeepening (Fig. S4c). We note, however, that data from drillings farther north^12^ disclosed a much thicker Quaternary suite of at least 270 m. However, since the thalweg is possibly sloping towards the north^12^, we actually do expect a lower thickness of the Quaternary suite in our section. Finally, our best model reaches a depth of 235 m (see below).

### Gravity modelling of the sedimentary infill of the Gürbe and the Aare valley with Prisma

**Figure 5:**
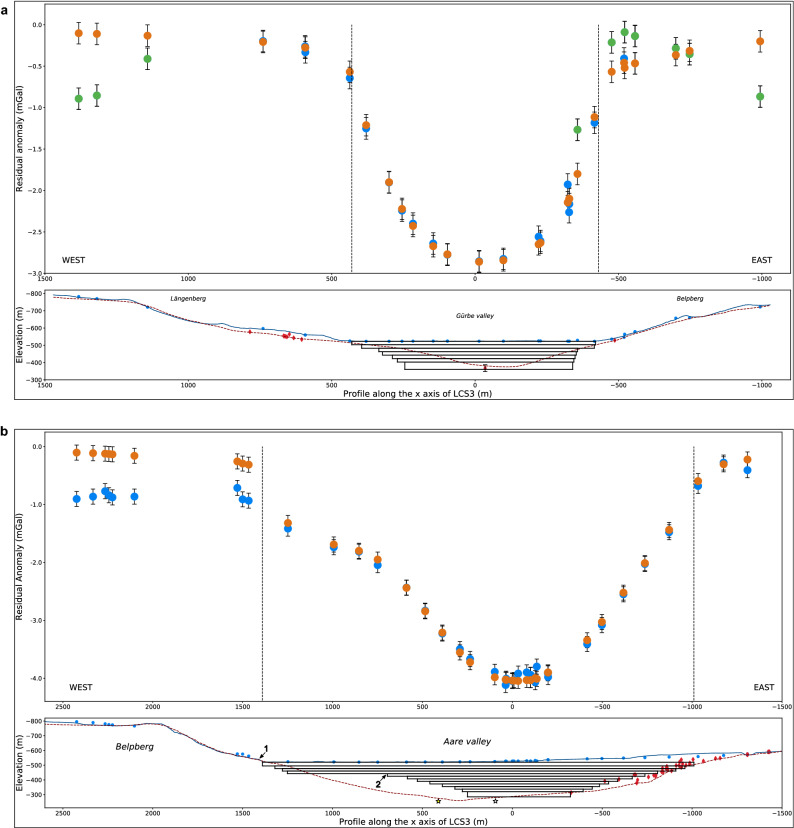
Results of gravity modelling. (**a**) Top: Final modelled residual anomaly, shown by the orange dots. Bottom: Plot of the final geometry of the Gürbe valley. This model suggests that the Gürbe valley is underlain by an overdeepening with an asymmetrical, U-shaped geometry. At our section, we estimate a maximum depth of c. 160 m. The eastern valley flank displays a knickzone at c. 40 m depth, which corresponds to the elevation where an LGM till has been encountered in the drilling RB9201 (Fig. 6b). (**b**) Similar plot for the Aare valley overdeepening, characterized by a maximum depth of c. 235 m. The western flank indicates the presence of two steps marked by the numbers 1 and 2 in the Figure. On the eastern margin, the bedrock flanks dips at c. 20°. The valley bottom has approximately the same cross-sectional width as the Gürbe overdeepening. Note that the drillings on the eastern margin imply a complex geometry as the bedrock depths vary along the overdeepening’s flank, and the overdeepening is turning counterclockwise, which complicates the modelling. Therefore, we proceeded through yielding a best fit between modelling results and drilling information through trial and error. Please refer to Fig. 4b,c for the legend and the explanation of the colours. Note, however, that the orange dots represent the modelled residual anomaly. Please refer to Fig. S2 for estimates of the density of the Molasse bedrock, and the supplementary note S4 for the determination of the density contrast between the Quaternary deposits and the Molasse bedrock. Intermediate modelling steps for assessing the bedrock geometry underneath the Gürbe and Aare valleys are illustrated and discussed in the supplementary notes S5 for the Gürbe valley, and S6, S7 for the Aare valley. The inputs and outputs of the final models, of both valleys, are available in the supplementary dataset Prisma.

For both overdeepenings, the modelling shows that a U-shaped cross-sectional geometry is consistent with the residual gravity anomalies across the valleys (Fig. 5). The modelling also illustrates that a V-shaped cross-sectional geometry yields a residual anomaly, which is lower than the observed values particularly on the flanks of the overdeepenings (Supplementary Figs. S5 and S6).

For the Gürbe valley overdeepening, the modelling shows asymmetric valley flanks. The western margin dips at c. 50°, whereas the eastern border is oversteepened and has a slope that dips at c. 80° (Fig. 5a). In addition, the eastern margin exhibits a knickzone. It separates a much flatter segment above it from a steep to over-steepened margin below it. This lower margin then dips at a nearly constant angle down to the overdeepening’s base. In contrast, the western margin shows a convex-up geometry where the dip continuously increases towards the base. The base itself is situated at a depth of c. 160 m and has a large lateral extent that is > 50% of the valley width, which points towards a rather flat geometry. Overall, the overdeepened valley can be characterized as a U-shaped cross-sectional geometry, which displays a composite shape with a broader upper part and a narrow trough at the base.

**Figure 6:**
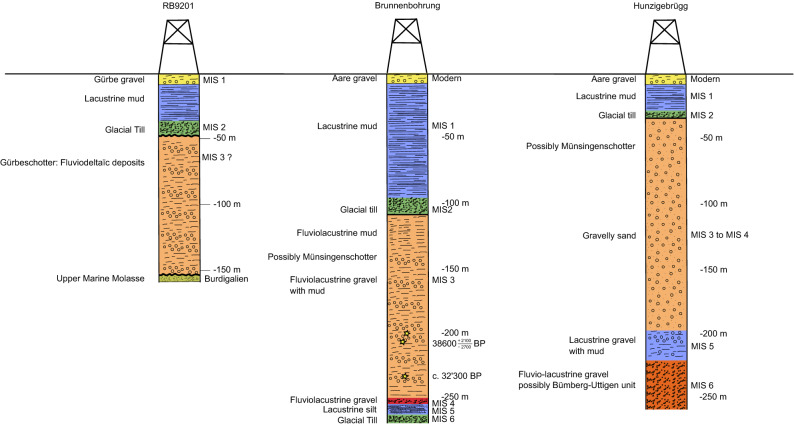
Sedimentary logs. Logs of one drilling in Gürbe valley^31,39^ and two drillings in Aare valley^26,33,36^. Information for drawing the log of the Aare valley is taken from the openly accessible database of the Canton Bern. For location of the drillings please refer to Figs. 1b and 2a,b. Different colours represent the various lithofacies.

The modelling of the Aare overdeepening also yields a U-shaped cross-section and an asymmetric geometry. This is mainly caused by the western margin, which shows two steps with distinct changes in the bedrock slope. The first step (Fig. 5b) occurs at the base of the Belpberg at an elevation between 540 and 530 m a.s.l. Below it, the bedrock dips at c. 20° until a depth level of c. 450 m a.s.l., where it flattens over a length of 700 m. This location is where the second step occurs (Fig. 5b), below which the bedrock steeply dips towards the base at a depth of 235 m, corresponding to an elevation of 285 m a.s.l. Interestingly, the second step is situated between the depth levels where the LGM moraine has been encountered in drillings (at c. 100 m depth in the Brunnenbohrung drilling and at c. 30 m depth in the Hunzigebrügg drilling, Fig. 6). On the eastern border, the overdeepening flank dips at c. 20° on average. The base of the Aare overdeepening is c. 600 m wide and nearly flat. Apart from these large-scale tendencies, some low residual gravity anomalies at the foothill of the Belpberg could not be fitted with the models. Similar to the Gürbe overdeepening, the Aare overdeepened valley can be characterized as a U-shaped cross-sectional geometry composed of a wider top segment and a narrower shape towards the base.

### Sedimentary architecture of the overdeepening fill encountered in the drillings

Three drillings, one in the Gürbe and two in the Aare, have been conducted close to our gravity section (Fig. 6). The stratigraphy in these drillings has been published in scientific journals and as reports in the database of the local authorities (see references below). Here, we present the results as stratigraphic logs and thereby re-interpret them in a harmonized way to facilitate a correlation between them.

The drillings showed that the bedrock underneath the target overdeepenings comprises the same Upper Marine Molasse (UMM) lithologies as the Belpberg^22,25,30,31^. In the Gürbe valley the drilling referred to as ‘Rotationsbohrung RB9201’ reached the UMM bedrock at the depth of 155 m (Fig. 6). A Quaternary sedimentary suite with silty gravel, referred to as the Gürbetalschotter^26,30,31^, follows atop the bedrock up to a depth level of 49 m. The ensemble of gravel and silt beds in these deposits, together with the occurrence of both rounded and angular clasts^31^, could point to the occurrence of an ice-contact or to a fluvio-deltaic environment^32^. The sequence is overlain by a ~ 5 m-thick diamict, which was probably deposited during the LGM c. 20,000 years ago. The uppermost 34 m comprises late-glacial clays deposited in a lake, and finally a ~ 8 m-thick sequence of fluvial gravel and overbank fines deposited by the modern Gürbe River^26,30,31^. Although we have no constraints on the age of the Gürbetalschotter unit, we tentatively assign an age between MIS 4 and 3 to this gravel, because these sediments show sedimentological similarities to a suite of gravel in the Aare valley that were encountered below a LGM till and that were dated with ^14^C to MIS 3 (see below).

In the Aare valley, a drilling referred to as ‘Brunnenbohrung Münsingen’ (Fig. 6) penetrated a 270 m-thick Quaternary suite without reaching the bedrock^33^. The sequence starts with a diamict, which is c. 6 m-thick and which was tentatively assigned to the ‘Riss’ glaciation^26^, possibly MIS 6. It is overlain by a c. 8 m-thick suite of interglacial lacustrine mud deposited between the ‘Riss’ and ‘Würm’ glacial periods^26^, which could correspond to the Eemian and thus to MIS 5e^34^. The overlying c. 4 m-thick gravel unit transitions into a ~ 120 m-thick suite of fluvio-lacustrine gravel up to a depth of 144 m, which was dated with ^14^C to c. 32,300 years BP (MIS 3) at three depth levels^33^. These gravel deposits are overlain by lacustrine mud up to a depth of 116 m. A c. 10 m-thick diamict is encountered between the depth interval of 116 and 96 m, which is then overlain by lacustrine mud up to a depth level of 8 m. While there are no age constraints for this diamict, it is likely a LGM deposit and thus of an MIS 2 age. The uppermost 8 m comprises gravel deposited by the modern Aare River. Approximately 10 km farther south, the lacustrine mud overlying the LGM diamict transition into fluvial deltaic gravel as reported in drillings^35^.

A further drilling, albeit destructive, was conducted at Hunzigebrügg c. 1.9 km north of our gravimetric section (Fig. 6). This drilling reached a depth of 264 m and did not encounter the bedrock. The basal c. 44 m-thick unit in this drilling comprises fluvio-lacustrine gravel, referred to as the Uttigen-Bümbergschotter^26^, was possibly deposited during the ‘Riss’ glacial cycle^36^ (MIS 6), or possibly before. It is overlain by a c. 26 m-thick suite made up of gravel and lacustrine mud, referred to as lacustrine sediments deposited between the ‘Riss’ and ‘Würm’ glacial periods^26^, which could correspond to the Eemian and thus to the MIS 5e^34,36^. The overlying c. 168 m-thick suite of gravelly sand, probably corresponding to the time span between MIS 4 and MIS 3, is overlain by a diamict deposited during the LGM. The uppermost 26 m comprise lacustrine mud and fluvial gravel of the Holocene.

Through these drillings, we find that the base of the LGM did not reach down to the base of the overdeepenings. The stratigraphic data also implies that the oldest deposits in our section have an age of MIS 6 or maybe older^36^, which is consistent with pollen records in a drilling (Thalgut) south of our section^37,38^. Furthermore, at this stage, there is no clear evidence that MIS 5d and MIS 4 glaciers^39^ significantly contributed to the shaping of the landscape in the region as distinct records are missing in the stratigraphy.

## Discussion

Although we successfully modelled the general pattern of the bedrock underneath both valleys, we are not able to model the low residual gravity anomalies at the foothill of the Belpberg in the Aare valley. We attribute this to the Quaternary sedimentary cover with a low density that forms two terrace levels at this location. Also in the Aare valley our modelled depth is slightly shallower than the depths the drillings farther north imply (Fig. 6) conforming the Reber and Schlunegger^12^ model where the thalweg of the Aare overdeepening is sloping towards the north. Nevertheless, our modelling results deviate from the bedrock topography model particularly on the flanks (red dashed line on Fig. 5a,b that shows the Reber and Schlunegger^12^ model). We explain this difference through a lack of drillings to constrain the continuation of the overdeepening margins at deeper levels, an aspect, which has also been noted before^12^. In fact, the Reber and Schlunegger^12^ model infers a progressive flattening of the valley flanks with depth, which however, is not confirmed by our results.

Despite these problems, our survey illustrates the strength of gravity modelling, which makes use of the fact that gravity is a field that provides very precise information on mass. Because gravity values (such as our residual gravity anomalies, Table S3) depend on both the volume (which includes the shape) and the density of the target geological bodies, interpretations are ambiguous, and independent constraints are needed as boundary conditions for the modelling. In particular, for the same gravity value along a profile, with no a-priori information on neither the density nor the geometry of the target bodies, multiple shapes, depths and densities could be considered as possible solutions. In our case, these hurdles have been avoided thanks to the Belpberg, which allows us to determine the density of the Molasse bedrock independent on the gravity modelling (Nettleton profiling^29^, see “Methods” section and Table S2). In addition, drillings that did not reach the bedrock provided constraints on the minimum values for the depth of the bedrock, whereas drillings that reached the bedrock on the lateral flanks (eastern margin of Aare valley) and the valley floor (Gürbe valley) provided first-order constraints for the overdeepenings’ geometries. Finally, we employed a combination of a high precision (2 m) digital elevation model (DEM) and global navigation satellite system (GNSS) measurements to precisely position the stations of interest and to retrieve exact values for the subsequent gravity correction (Table S1). Data measured at a point provides information on the density variation and thickness differences between geological bodies in three dimensions (3D). Some flexibility in designing a survey is thus possible, particularly where the environment does not allow a strict alignment of stations and a constant spacing between them. However, because the gravity effect decreases exponentially with distance, we lose resolution the farther we are situated from the objects of interests. In this context, with regards to the Gürbe valley, most of the stations of the gravimetric map of Switzerland^27^ have a spacing that is too large for the contribution of the overdeepening gravity to be recorded (Fig. 3). Since the spacing of our stations is much closer, we can measure the gravity signal of the Gürbe overdeepening at a high resolution (Fig. 4b). Moreover, the uncertainty of the a-priori information at depth is greater than at the surface, with the consequence that the distance between the stations can be adjusted according to the targeted geological feature. In our case, because the overdeepening flanks contain most of the information pertinent for inferring the glacial processes at work (see below), we selected a short spacing of 50 to 100 m between the survey stations at these flanks.

Our gravity modelling shows that a U-shaped cross-sectional geometry for the overdeepenings is consistent with the residual Bouguer anomalies measured across the valleys (Fig. 5), and that a V-shaped cross-sectional geometry yields a residual gravity anomaly, which is significantly lower than the observed values particularly on the flanks of the overdeepenings (Supplementary Figs. S5 and S6). This U-shape suggests that the formation of the overdeepenings was most likely accomplished through glacial carving^3,40^. U-shaped valley geometries have indeed been considered as indicative for glacial shaping provided that the corresponding area has not experienced high uplift rates^41^, as is the case for the Swiss Plateau^42^. Further supporting evidence for the inferred occurrence of glacial carving is provided by the flank’s oversteepening, which is particularly strong on the eastern margin of the Gürbe valley’s trough (Fig. 5a). We attribute this asymmetry to the inward shift of the shear stress centre line of a glacier making a bend in the downstream direction^43^. Such a shift would enhance glacial erosion through abrasion on the inner flank of the curve in response to an accumulation of stress, which has been used to explain oversteepened bedrock flanks in a modern setting^44^. Finally, the occurrence of glacial carving has also been inferred from a drill core in the Bern area close to our survey location. At this site(Rehhag), the occurrence of a glacial till atop the bedrock together with structures of glacio-tectonic deformation has been used to propose that glacial carving was an important erosional process^28^. We note, however, that it is not possible at this stage to interpret whether the valleys were originally formed by fluvial dissection and then widened by glacial carving^18,45^.

Erosion by overpressurized meltwater could have occurred together with the glacial carving, specifically for the flushing of erosional products. Such a mechanism has also been considered to contribute to the shaping of many overdeepenings, particularly where adverse slopes are found farther downstream^12–14,17^. Furthermore, it was documented^11^ that bedrock fracturing through subglacial water pressure may have contributed to the deep glacial carving of an overdeepening in north-eastern Switzerland, similarly leading to U-shaped valleys^20^. Although we lack distinct geomorphic features in the bedrock that are diagnostic for erosion by subglacial meltwater (such as, e.g., potholes, channels, and fractured rocks^11,13,46^), we cannot exclude the possibility that glacial hydrological processes were contributing to the carving of the bedrock in the study area^17^.

**Figure 7:**
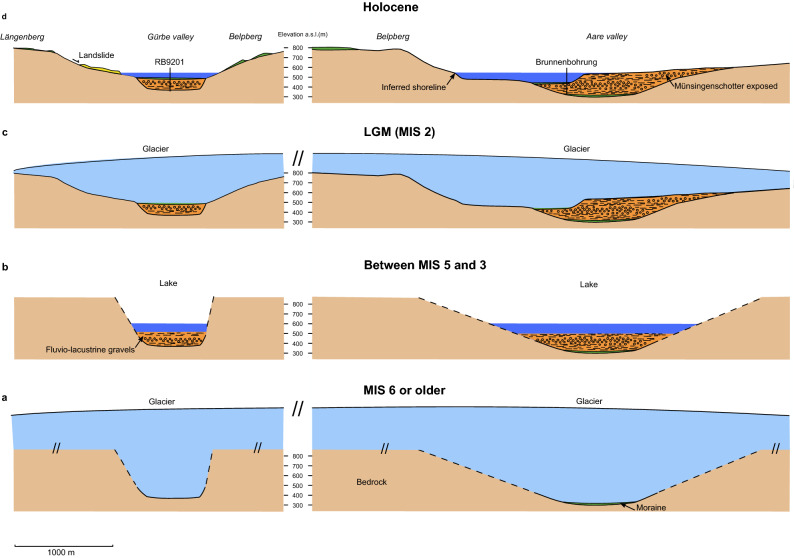
Evolution. Summary figure showing the evolution of the overdeepenings with various time steps in response to glacial carving (**a**) during MIS 6 or previous glaciations, (**b**) during the time between MIS 5 and MIS 3, (**c**) during the LGM time, and (**d**) during the Holocene. Please note that stage (**b**) illustrates a lacustrine phase with a lake level of (**c**). 600 m a.s.l,. The lake was most likely filled by gravel and lacustrine mud up to this elevation. The corresponding sediments are currently exposed as Münsingenschotter (Fig. 2a). This gravel unit then formed the basis of the LGM glacier as illustrated in (**c**). The early Holocene was characterized by a lacustrine phase where wave erosion made the final adjustments particularly on the eastern flank of the Belpberg. This period was also characterized by landslides particularly on the western flank of the Gürbe valley. Currently, the Aare and Gürbe valleys are occupied by streams.

The stratigraphic information suggests that the base of the LGM glacier was situated at depth levels between 30 and 100 m below today’s surface and did not reach the bedrock (Fig. 6). The drilling information also suggests that the basal part of both overdeepenings was likely carved by one or multiple glaciers during MIS 6 and possibly before. It further suggests that glaciers that advanced from the Alps to the Swiss Plateau during MIS 4 and MIS 5d times^39^ did not extend as far north as our cross-section, because no distinct glacial diamict of a related age has been encountered. Since moraines of a post-LGM age^22–24^ are still well preserved on the lateral flanks of both valleys and on top of the Belpberg, we can infer that the exposed bedrock above the valley floors largely reflects the bedrock geometry that framed the flow of the LGM glacier on its lateral margins. Thus, the composite shape of the overdeepenings (Fig. 5) can be attributed to an older narrow and (over)steep(ened) carving during MIS 6 and a re-activation during the LGM, leading to a shallow and broader upper part (Fig. 7). This difference in glacial incision could be related to the different lithologies where the MIS 6 and possibly previous glaciers mainly needed to carve into tender and porous Molasse sandstones^47,48^, whereas the LGM glacier would have to cut into more competent, cohesive glacio-lacustrine marls^28^. Alternatively, these contrasts in glacial deepening could also be explained by the differences in the thicknesses between the LGM and the MIS 6 or older glaciers. In particular, it was reported that erratic boulders, which were dated to MIS 6 with in-situ cosmogenic ^10^Be, are situated along the Jura Mountains c. 40 km north of our study area, and that these boulders occur at elevations c. 200–300 m higher than those assigned to the LGM glaciation^49^. This implies that the MIS 6 glaciers were most likely thicker than the LGM ice sheet at least in the Swiss Plateau^49^. Because glacial erosion depends on both the velocity and the shear stress of ice^50,51^, which is most likely also valid for ice forming overdeepenings^17^, a thick glacier would have a greater ability to erode the bedrock than a thin ice sheet. This is the case because the ice thickness influences both the velocity of the moving glacier at an exponent between 1 and 2^50^, and the shear stress at its base with a linear relationship. On the lateral flanks, however, the erosive power of a glacier is mainly dependent on the flow velocity. If these inferences are valid, then lateral erosion scales to ice thickness by an exponent between 1 and 2, whereas the carving at the base scales with the ice thickness with an exponent > 2. Accordingly, we propose a scenario where a thick MIS 6 (or possibly older) glacier resulted in the formation of an overdeepening with a flat and deep base in combination with steep lateral flanks (Fig. 7). This trough was then partially or completely filled with fluvio-lacustrine sediments as revealed by the sedimentary suite. The subsequent LGM glacier with a presumably lower thickness between 400 and 500 m^21^ followed the previously sculpted valley, widened it but did not carve through the entire sedimentary fill.

In the Aare valley, the bedrock topography model of Reber and Schlunegger^12^ shows the occurrence of a shoulder at the base of the Belpberg (step 1 on Fig. 5b, and Fig. 7d). As mentioned before, the elevation of this shoulder nearly coincides with that of the terminal moraine of the Aare glacier that was dated to 19,000 years BP^23^. This terminal moraine may have built the dam of a lake that extended at least 20 km farther south as inferred from lacustrine mud found in drillings (Fig. 6). We thus interpret this shoulder to reflect a shoreline of the lake before it was filled with lacustrine mud.

In summary, gravity data in combination with available stratigraphic information allows us to develop a scenario of how the landscape in the study area has changed at least since the Late Pleistocene. During the time of the greatest ice extent, which possibly occurred during the MIS 6 or ‘Riss’ glaciation (or before^52^), large and thick glaciers were carving a deep and relatively narrow trough into the Gürbe and Aare valley regions (Fig. 7a). The subsequent times, possibly spanning MIS 5 through MIS 3 (Fig. 7b) experienced a phase with a large lake in both valleys with a lake level at or lower than c. 600 m a. s. l.^28^. This lake was filled with fluvio-lacustrine gravel, some of which are exposed on the eastern margin of the Aare valley (Münsingen or Uttigen-Bümberg Schotter; Figs. 2a, 7d) and have been encountered in drillings (Fig. 6). During the LGM when the ice shield in the Bern region reached a surface elevation of c. 1000 m a. s. l.^21^, the Belpberg and Längenberg obtained their modern shapes (Fig. 7c). Our reconstructions imply that in contrast to the previous large glaciations, the LGM glacier was mainly widening the valleys. Finally, the Holocene started with a lacustrine period (Fig. 7d), and waves were forming a step at the eastern foothill of the Belpberg (see arrow on Fig. 7d). The Gürbe and Aare valleys were finally filled with the fluvial gravel of the modern Gürbe and Aare Rivers.

As a conclusion, gravity profiling in combination with stratigraphic data yields constraints on the glacial history of a region of interest and offers insights into the mechanisms for the formation of overdeepenings. In the case of the bedrock troughs in Central Switzerland, we infer that these overdeepenings were mainly formed by glacial carving, where ice thickness is likely the main driving parameter. Additionally, because these bedrock troughs hosted paleolakes, waves also contributed to the bedrock shaping at least during the latest stage of a glacial cycle.

## Methods

### Landscape morphometry and compilation of existing gravity information

Topographic cross-sections were extracted from the 2 m-resolution SwissAlti3D DEM of swisstopo. The bedrock geology map^22,25^ is taken from the openly accessible swisstopo database. The bedrock topography model underneath the target overdeepenings, which is based on drillings, has been extracted from the openly accessible digital database of the Canton Bern, Switzerland (geo.apps.be.ch). We compiled existing gravity information for the area surrounding the study region from the gravimetric map of Switzerland. The subtraction of the bedrock topography from the surface topography yields information on the thickness pattern of the Quaternary sediments. Such maps have already been published before^12–14^, and we refer the reader to these articles.

### Gravity survey

We conduced gravity measurements with the swisstopo gravimeter CG5 (precision of 0.001 mGal). It relates the measurements to the calibrated base at the Swiss Metrological Institute, 2,601,930.7/1,197,021.8 (Swiss coordinate system LV95). At each location, we repeated the measurements 8 times until the variation between the last three acquisitions were < ± 0.001 mGal. Each of these measurement cycles lasted either 30 or 45 s when gravity data was collected > 250 times. The results were returned as the mean value (Supplementary Data Table S1). The coordinates were determined using a Leica GNSS (precision of < 1 cm in the x- and y-directions, and c. 10 cm in the z-direction).

We conducted the survey on a section nearly perpendicular to the orientation of the overdeepenings and the Belpberg^12^. We selected 82 sites with a spacing of c. 50 and 300 m. Approaching the Belpberg’s flanks, the measurement sites, referred to as stations, were closer to each other (spacing < 100 m) to retrieve more information on the bedrock surface beneath the valley border. Where possible, we aligned the stations within a 100 m-wide bandwidth.

The gravimeter was placed on a plane surface with no pronounced local topography (steps, flanks, border of cliffs, inferred underground infrastructure and building). We avoided measurements during strong wind because this could bias the measurements. At each site a series of individual measurements of gravity and instrument tilt were performed to check for disturbances from seismic events. If the individual values were constant within the instrument accuracy over several minutes, the observed gravity value was calculated from the average of the best 4–5 measurements out of 8, evaluated by the swisstopo software GRAVNIV (Gravity for Nivellement).

We observed an offset of 0.735 mGal between our gravity results and the openly accessible gravimetric map of Switzerland, because first, we used the GRS80 ellipsoid for the normal gravity formula while the GRS67 formula was employed for the calculation of the values for the gravimetric map of Switzerland, yielding in 0.854 mGal higher gravity values. Second, we used the ETRS89 latitude while the gravimetric map was calculated based on the local Swiss Bessel ellipsoid. The corresponding shift of 4.75 arc seconds (c. 146 m) to the north compared to our reference thus results in a lower gravity value offset by 0.119 mGal.

We conducted the Bouguer anomaly corrections (Table S1) using state-of-the art Eqs.^53,54^ that are implemented in the swisstopo software Quawirk. The corrections consider 7 components: latitude, elevation, tides, atmospheric pressure, a contribution of the geological units between the stations and the surface of the ellipsoid, curvature of the Earth, and a gravity contribution of the topography.

The two main sources of errors are the precision of the elevation and the local gravity measurement. The latter was obtained by a series of individual measurements (see above). Measurements affected by passing seismic waves from earthquakes were identified as real or possible outlier observations (4 out of 82) and deleted from the data set. This finally yielded 78 stations, the results of which were used for further analyses and modelling (Table S1). For a realistic uncertainty estimate of the Bouguer gravity observation, we re-measured the gravity at 11 sites along the profile and within less than 10 m distance from the original location. The results confirm an overall uncertainty of ± 0.13 mGal for the Bouguer values along our profile (Supplementary Table S1).

### Estimation of the bedrock density by the Nettleton method

The Belpberg has a linear elongation with a nearly symmetric cross-sectional geometry and is made up of a monotonous suite of UMM sandstones. Therefore, this mountain ridge offers ideal conditions for determining the bedrock density with the Nettleton method^29^. Accordingly, we calculated the Bouguer anomaly with a reference height of 520 m a.s.l., and we used the Bouguer gravity values of the stations between the eastern side of the Gürbe valley and the western side of the Aare valley across the Belpberg (Fig. 4a). The selection of this reference elevation allows us to explicitly calculate the gravity effect of the Belpberg topography for different bedrock density values. We calculated the Bouguer anomalies for the section across the Belpberg and iteratively increased the bedrock densities from 2300 kg/m^3^ to 2670 kg/m^3^ with 100 kg/m^3^ increments (Fig. S2; Table S2). The analysis yielded a density of 2500 kg/m^3^ for the Upper Marine Molasse unit that comprises the bedrock beneath the Gürbe and the Aare valley along our profile.

### Development of Prisma for estimating the geometry of the overdeepening fill

We developed an open-source software (Prisma), which allows to calculate the three-dimension gravity effect of a disturbing body, such as an overdeepening fill, on a station. This requires an input for the density of both the body to be modelled (in our case the overdeepening) and of the environment (the bedrock). The output of the model is a table containing information on the stations (location, elevation), and the calculated residual anomaly for each of them.

This software is written in Python 3 and designed as a forward modelling approach. It is based on analytical solutions^55^. Note that the Nagy Eq.^55^ uses arcsine functions to account for the vertical gravity component. However, this solution fails to properly account for cases where a negative value for the arcsine has to be considered^56,57^. We use an arctan term instead^56,57^, which we implemented in our routine.

The routine calculates the gravity effect on a station caused by right-angled prisms, approximating the geometry of the target body. Note that instead of employing existing 3D modelling software packages^58–60^, we constructed a code^55,61^ (Fig. S3) that is tailored to model the gravity effect of the target structure of this study thereby considering the modelling situation at hand (a-priori information and main structural parameters of interest, oblique profiles sampled by dispersed gravity stations). In addition, since the nature of glacial carving can be best explored when quantifying the geometry of the lateral flanks of an overdeepening^44^ (which is the scope of this work), a cross-sectional survey in combination with a 3D gravity model is a suitable strategy for such an exploration.

### Estimation of density contrast between Molasse bedrock and overdeepening fill

We used state-of-the-art approaches^18^ to determine the average density contrast between the bedrock (2500 kg/m^3^) and the Quaternary sediments, and we used the maximum value of the residual anomalies as benchmark^18^. We thus defined a prism for the Prisma routine, which includes the surface outlines of the overdeepening (Fig. S4). We iteratively increased the prisms’ thicknesses from 50 to 300 m by 50 m steps in-between. During each iteration, we assigned density contrasts between 100 and 600 kg/m^3^ with 100 kg/m^3^ intervals, which includes the c. 2200 kg/m^3^ density value measured for highly consolidated Quaternary sediments close to Bern^28^. We followed the same approach for the Aare valley^18^, but with a slightly modified initial setup (Fig. S4), which is justified because of the greater width and depth of the overdeepening fill^12^. We thus started with a thickness of 160 m, which we iteratively increased up to 300 m with 20 m-thick intervals. For both the Gürbe and the Aare valleys, we calculated the residual gravity anomaly for a single station recording the largest residual anomaly, which is located in the centre of each valley (i.e., station 1015 for the Gürbe valley, and station 3011 for the Aare valley). Because the maximum residual anomaly is known for both valleys (Fig. 4), we determined the thickness-density contrast ensemble that yields the closest similarity to the observed data^18^.

### Modelling the cross-sectional geometry of the overdeepenings

As a next step, we refined our models using the routine Prisma by (i) increasing the number of prisms for different layers, (ii) testing U- versus V-shaped valley flanks, and (iii) adjusting the prisms’ widths and the slopes of the flanks independently. By a succession of multiple models, we finally approached a best fit to the observations. We accepted a final model where the calculated residual anomaly at each station fits the measured anomaly within less than the uncertainty. We accepted exceptions from this principle for stations where the distance to the profile was > 100 m for the Gürbe valley. We justify this approach, because the valley geometry is not fully two-dimensional.

## Supplementary Information

Supplementary Information 1.

Supplementary Information 2.

Supplementary Information 3.

Supplementary Information 4.

Supplementary Information 5.

## References

[1] Häberli W (2016). On the morphological chracteristics of overdeepenings in high-mountain glacier beds. Earth Surf. Process. Landf..

[2] Patton H (2016). Greenland and Antarctic ice sheets: Implications for overdeepening origin and evolution. Quat. Sci. Rev..

[3] Magrani F, Valla PG, Gribenski N, Serra E (2016). Glacial overdeepenings in the Swiss Alps and foreland: Spatial distribution and morphometrics. Quat. Sci. Rev..

[4] Jørgensen F, Sandersen PBE (2006). Buried and open tunnel valleys in Denmark—erosion beneath multiple ice sheets. Quat. Sci. Rev.

[5] Powell RD, Khalil Moh’d B, Masri A (1994). Late Ordovician-Early Silurian glaciofluvial deposits preserved in palaeovalleys in South Jordan. Sed. Geol..

[6] Douillet D (2012). Late Ordovician tunnel valleys in southern Jordan. Geol. Lond. Spec. Publ..

[7] Jordan P (2010). Analysis of overdeepened valleys using the digital elevation model of the bedrock surface of northern Switzerland. Swiss J. Geosci..

[8] Preusser F, Reitner JM, Schlüchter C (2010). Distribution, geometry, age and origin of overdeepened valleys and basins in the Alps and their foreland. Swiss J. Geosci..

[9] Burschil T (2019). Unravelling the shape and stratigraphy of a glacially-overdeepened valley with reflection seismic: The Linz Basin (Austria). Swiss J. Geosci..

[10] Buechi M (2018). Multiple Quaternary erosion and infill cycles in overdeepened basins of the northern Alpine foreland. Swiss J. Geosci..

[11] Gegg L (2020). Brecciation of glacially overridden paleokarst (Lower Aare Valley, northern Switzerland): Result of subglacial water-pressure peaks?. Boreas.

[12] Reber R, Schlunegger F (2016). Unravelling the moisture sources of the Alpineglaciers using tunnel valleys as constraints. Terra Nova.

[13] Dürst Stucki M, Reber R, Schlunegger F (2010). Subglacial tunnel valleys in theAlpine foreland: An example from Bern, Switzerland. Swiss J. Geosci..

[14] Dürst Stucki M, Schlunegger F (2013). Identification of erosional mechanismsduring past glaciations based on a bedrock surface model of the central European Alps. Earth Planet. Sci. Lett..

[15] Rosseli A, Olivier R (2003). 2.5D gravimentric modelling and a 1:100’000 isohypse map of the rocky substrate of the Rhone valley between Villeneuve and Brig (Switzerland). Eclogae geol. Helv..

[16] Cook SJ, Swift DA (2002). Subglacial basins: Their origin and importance in glacial systems and landscapes. Earth Sci. Rev..

[17] Herman F (2011). Glacial hydrology and erosion patterns: A mechanism for carving glacial valleys. Earth Planet. Sci. Lett..

[18] Kissling E, Schwendener H (1990). The Quaternary sedimentary fill of some Alpine valleys by gravity modeling. Eclogae geol. Helv..

[19] Perrouty S (2015). Geometry of two glacial valleys in the northern Pyrenees estimated using gravity data. Comptes Rend. Geosc..

[20] Nagra. 2D seismic exploration and provisory interpretation of overdeepened Quaternary valleys around the Nagra siting regions Zürich Nordost and Nördlich Lägern. Arbeitsbericht NAB 18–22, 51 pp., Nagra, Wettingen (2018).

[21] Bini, A., et al. Die Schweiz während des letzteiszeitlichen Maximums (LGM) 1:500'000. Bundesamt für Landestopografie swisstopo, Bern, Switzerland (2009).

[22] Spicher, A. Geologische Karte der Schweiz 1:500’000. *Schweiz. Geol. Komm.*, Bern, Switzerland (1972).

[23] Wüthrich L (2018). 10Be surface exposure dating of the last deglaciation in the Aare valley. Switzerland. Swiss J. Geosci..

[24] Meichtry, N. *Last Deglaciation of the Aare Valley*. Ms Thesis, Univ. Bern, Switzerland, 76 pp. (2017).

[25] Beck, P. & Rutsch, P. Geologische Karte der Schweiz, Kartenblatt Münsingen-Konolfingen-Gerzensee-Heimberg. *Schweiz. Geol. Komm.*, Bern, Switzerland (1949).

[26] Kellerhals P, Isler A (1983). Profilserie durch die Quartärfüllung des Aare- und des Gürbetroges zwischen Thunersee und Bern. Eclogae geol. Helv..

[27] Olivier, R., Dumont, B. & Klingele, E. Carte gravimétrique de la Suisse (Anomalies de Bouguer) 1:500’000. Bundesamt für Landestopographie swisstopo (2008, 2011).

[28] Schwenk, M., Schlunegger, F., Gribenski, N., Schläfli, P., Bandou, D., Douillet, G., Krbanjevic, J. Stratigraphic and Multi Scanner Core Logging (MSCL) data plus supplementary luminescence dating material obtained from the scientific drilling QDR-RE-IfG and its drill site in the Aare Valley, Bern CH. GFZ Data Services (2021). 10.5880/fidgeo.2021.021.

[29] Nettleton LL (1939). Determination of density for the reduction of gravimeter observations. Geophyscics.

[30] Soom, M. Belp, Belpmoos, Bohrung RB 9301. WEA des Kantons Bern, WEA Ord.-Nr. 603/195.5, Anhang 8, 3 pp. (1993).

[31] Geotest. Grundlagen für Schutz und Bewirtschaftung der Grundwasser des Kantons Bern. Hydrogeologie Gürbetal und Stockental. Wasser- und Energiewirtschaftsamt des Kantons Bern WEA, 123 pp. (1995).

[32] Lønne I (1995). Sedimentary facies and depositional architecture of ice-contact glaciomarine systems. Sediment. Geol..

[33] Kellerhals, P. & Haefeli, C. Brunnenbohrung Münsingen. Geologische Dokumentation des Kantons Bern, WEA-Geologie, Beilage Nr. 2, 7 pp. (1984).

[34] Schläfli P (2021). Palynological investigations reveal Eemian interglacial vegetation dynamcis at Spiezberg, Bernese Alps, Switzerland. Quat. Sci. Rev..

[35] Gruner U (1993). Eiszeitliche Trogbildung im Raum Bern. Mitt. Natf. Ges. Bern.

[36] Zwahlen P, Tinner W, Vescovi E (2021). Ein neues EEM-zeitliches Umweltarchiv am Spiezberg (Schweizer Alpen) im Kontext der mittel- und spätplesitozänen Landschaftsentwicklung. Mitt. Naturf. Ges. Bern.

[37] Welten, M. Pollenanalytische Untersuchungen im Jüngeren Quartär des nördlichen Alpenvorlandes der Schweiz. Beitr. Geol. Karte Schweiz, NF 156, Schweiz. Geol. Komm., Bern, 210 pp. (1982).

[38] Welten, M. Neue pollenanalytische Ergebnisse über das Jüngere Quartär des nördlichen Alpenvorlandes der Schweiz (Mittel- und Jungpleistozän). Beitr. Geol. Karte Schweiz, NF 162, Schweiz. Geol. Komm., Bern, 52 pp. (1988).

[39] Preusser F (2011). Quaternary glaciation history of Northern Switzerland. Quat. Sci. J..

[40] Temovski M (2018). Glacial geomorphology and preliminary glacier reconstruction in the Jablanica Mountain, Macedonica, Central Balkan Peninsula. Geosciences.

[41] Prasicek G, Larsen IJ, Montgomery DR (2015). Tectonic control on the persistence of glacially sculpted topography. Nat. Commun..

[42] Kahle HG, Pfiffner OA, Lehner P, Heitzmann P, Müller S, Steck A (1997). Recent crustal movements, geoid and density distribution: Contribution from integrated satellite and terrestrial measurements. Results of the National Research Program 20 (NRP 20).

[43] Echelmeyer K, Kamb B (1987). Glacier flow in a curving channel. J. Glaciol..

[44] Nishiyama R (2019). Bedrock sculpting under an active alpine glacier revealed from cosmic-ray muon radiography. Sci. Rep..

[45] Harbor JM (1992). Numerical modeling of the development of U-shaped valleys by glacial erosion. GSA Bull..

[46] Sudgen DE, Denton GH, Marchant DR (2017). Subglacial meltwater channel systems and ice sheet overriding, Asgard Range, Antarctica. Geogr. Ann. Ser. A Phys. Geogr..

[47] Platt NH, Keller B (1992). Distal alluvial deposits in a foreland basin setting—the Lower Freshwater Miocene), Switzerland: Sedimentology, architecture and palaeosols. Sedimentology.

[48] Kühni A, Pfiffner OA (2001). The relief of the Swiss Alps and adjacent areas and its relation to lithology and structure: Topographic analysis from a 250-m DEM. Geomorphology.

[49] Graf A (2015). Multiple advances of Alpine glaciers into the Jura Mountains in Northwestern Switzerland. Swiss J. Geosci..

[50] Herman F (2015). Erosion by an Alpine glacier. Science.

[51] Cook S (2020). The empirical basis for modeling glacial erosion rates. Nat. Comm..

[52] Schlüchter, C. Akçar, N., Ivy-Ochs, S. The Quaternary Period in Switzerland. In *Landscapes and Landforms of Switzerland*. (ed. Reynard, E.) 47–69 (World Geomorphological Landscapes, Cham, Springer, 2021). 10.1007/978-3-030-43203-4_4.

[53] Hinze WJ (2005). New standards for reducing gravity data: The North American gravity database. Geophysics.

[54] Klingele, E. & Schwendener, H. Geophysical investigation programme of Northern Switzerland: Gravimetric measurements 81/82. NAGRA-NTB 84–22, 60 pp. (1984).

[55] Nagy D (1966). The gravitational attraction of a right rectangular prism. Geophyscis.

[56] Banerjee B, DasGupta SP (1977). Gravitational attraction of a rectancular parallelepiped. Geophysics.

[57] Karcol P, Pašteka R (2019). On the two different formulas for the 3D rectangular prism effect in gravimetry. Pure Appl. Geophys..

[58] Price, A. FastGrav. http://fastgrav.com, Accessed 14 December 2021 (2018).

[59] Uieda, L., Oliveira Jr, V. C. & Barbosa V. C. F. Modeling the Earth with Fatiando a Terra. In *Proceedings of the 12th Python in Science Conference* 91–98 (2013).

[60] Schmidt, S., Anikiev, D., Götze, H. J., Gomez Garcia, À., Gomez Dacal, M. L., Meeßen, C., Plonka, C., Rodriguez Piceda, C., Spooner, C., & Scheck-Wenderoth, M. IGMAS+-a tool for interdisciplinary 3D potential field modelling of complex geological structures. In *EGU General Assembly Conference Abstracts* 8383 (2020).

[61] Talwani M (1973). Computer usage in the computation of gravity anomalies. Methods Comput. Phys..

