# Supplementary material for: Three-dimensional gravity modelling of a Quaternary overdeepening fill in the Bern area of Switzerland discloses two stages of glacial carving
Source: Sci Rep. 2022 Jan 27;12:1441. doi: 10.1038/s41598-022-04830-x (PMC8795381; doi:10.1038/s41598-022-04830-x)
Supplement: Supplementary file 4 — Supplementary Information 4. [file 41598_2022_4830_MOESM4_ESM.pdf]

Supplement for Three-dimensional gravity modelling of a Quaternary overdeepening fill in the Bern area of Switzerland discloses two stages of glacial carving

D. Bandou, F. Schlunegger, E. Kissling , U. Marti, M. Schwenk, P. Schläfli, G. Douillet, D. Mair

S1: Location of sections and prisms

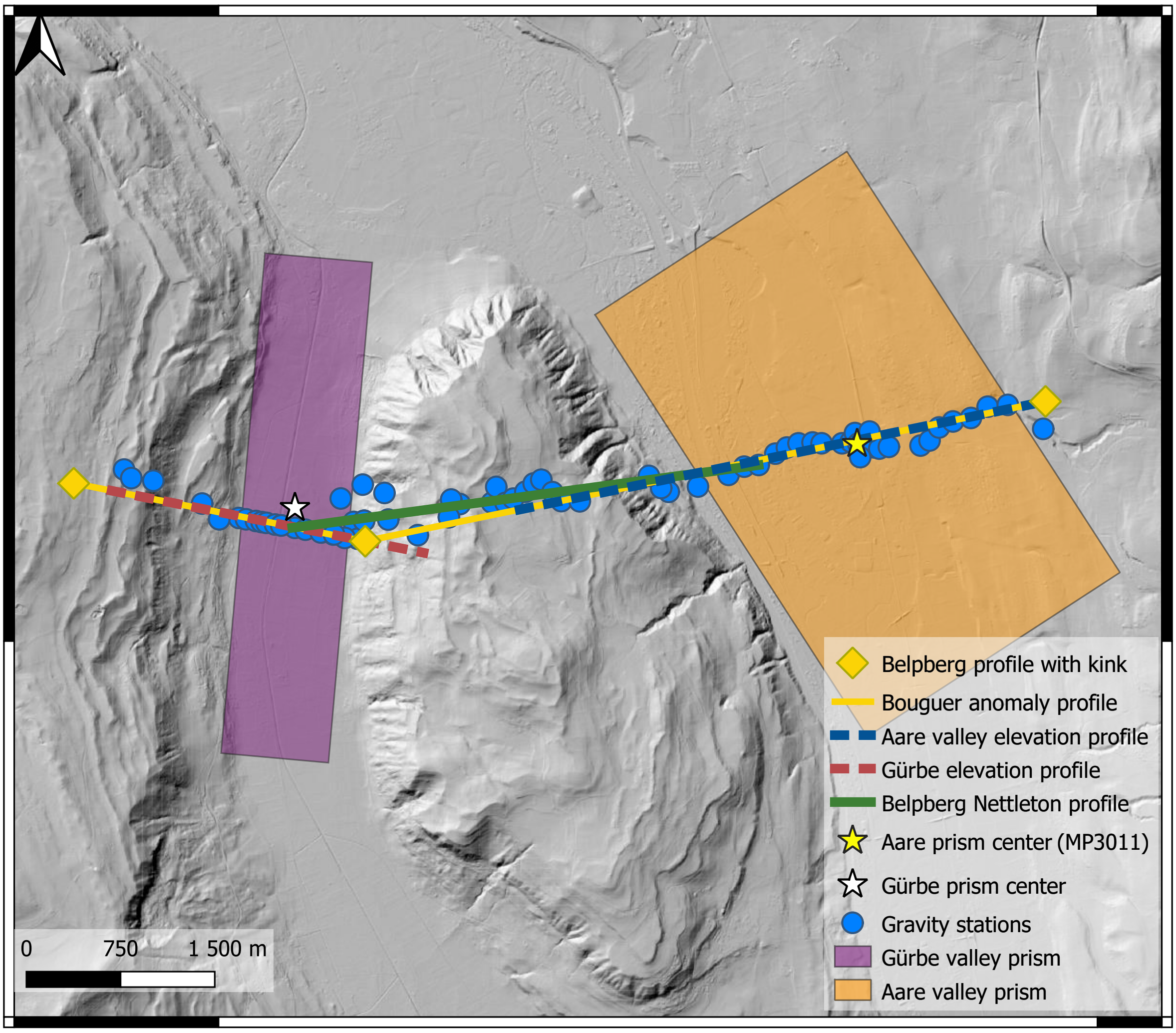

Figure S1: Map showing the location of the different sections used for the calculation of the Bouguer anomaly, the Nettleton profile, and the residual anomalies of the Gürbe and Aare valleys. See following sections for justification of location of selection. The stars represent the prism centres and the rotation centres. This figure and the following ones have been drafted in QGIS 3.12.0 (openly accessible: https://www.qgis.org/), the DEM is openly accessible (SwissAlti3D, © swisstopo).

S2: Estimation of the density of the Upper Marine Molasse bedrock using the Nettleton method

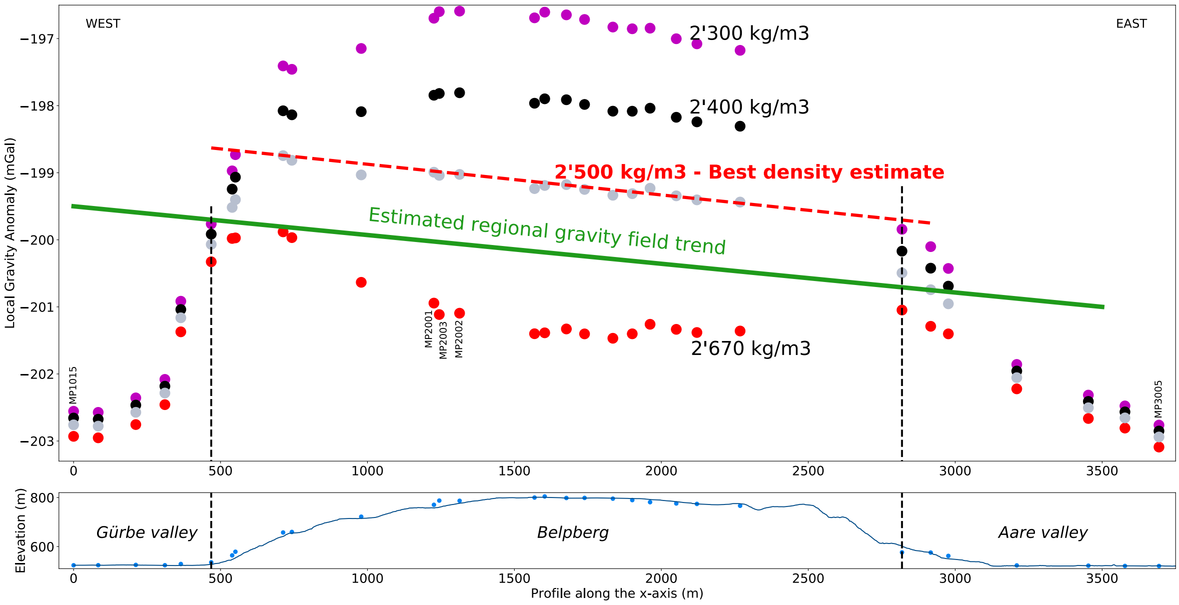

Figure S2: Nettleton profile for the Belpberg. The trend line of the local gravity anomaly for the bedrock density of 2500 kg/m^3 parallels the regional gravity field trend. This suggests that the density value, for which there is the least correlation with the topography, is the best estimate for the bedrock density. The westernmost station is 1015 and the easternmost station is 3005 (Table S1). See figure 3 (main text) for location of stations, and figure S1 for location of section. The triplet stations 2001, 2003 and 2002 between 1000 m - 1500 m distance show a local effect related to moraines.

Because the Belpberg comprises a homogeneous suite of UMM sediments[1,2] (see main text), we designed our elevation profile (Figure S1) such as that it is oriented as much perpendicular as possible to the structure of the Belpberg while remaining parallel to the stations. We assigned densities between 2300 kg/m^3 and 2670 kg/cm^3 to the Upper Marine Molasse bedrock, the latter of which is the standard density commonly used in gravity research[3]. We calculated the resulting Bouguer anomalies for the target section across the Belpberg and iteratively increased the bedrock densities with 100 kg/m^3 increments. The topography correction for the Bouguer anomaly was obtained with two DEMs, the selection of which depends on the distance from the station. The Swisstopo 2 m DEM is used up to a distance of 200 m, while the 25 m EuroDEM was used for larger distances between 200 m and 167 km. Accordingly, the size of the DEM cells and the calculation method also depends on the distance to the station:

-200 m to 2 km: 25 m with prisms inclined at the topography surface.

-2 km to 20 km: 50 m DEM as mass lines.

-20 km to167 km: 500 m DEM as mass lines.

We proceeded with these calculations until we found a best fit between the trend of the Bouguer anomalies across the Belpberg and the regional trend (Figure S2). In particular, the Bouguer anomalies that are based on a bedrock density of 2670 kg/m^3 yields an

inverse shape of the surface topography, suggesting that the density of the Upper Marine Molasse bedrock is lower than that. In contrast, the consideration of a density of 2400 kg/m^3 yields a positive correlation, implying that this density yields in an underestimation of the gravity contribution of the Upper Marine Molasse bedrock. We finally found a best fit between the trend of the Bouguer anomalies across the Belpberg and the regional trend if we consider a density of 2500 kg/m^3 for the Upper Marine Molasse bedrock (Figure S2). Note that there is a very local anomaly between points MP2001, MP2003 and MP2002 where we estimate a local density of 2450 kg/m^3 as indicated by the concave and convex triplet anomalies for density values of 2400 kg/m^3 and 2500 kg/m^3, respectively. This suggests the occurrence of missing mass in a relative sense, which we attribute to the effect of moraine ridges in this region[1,2].

S3: Development of the gravity modelling software package PRISMA

We developed a routine (see Python code in Supplementary Data referred to as Prisma) to address two major goals including (i) the assessment of the bulk densities of the Quaternary infill and (ii) the determination of the cross-sectional geometry of the overdeepenings based on 3D a-priori information that is already available (Figure S4a)[4]. To achieve these goals, we developed a code referred to as Prisma (Figure S3 and Prisma in Supplementary Data). Prisma calculates the gravity effects of right-angled prisms with a given density on a station. It is thus used to calculate the gravity effect of simple structures, quarries and mountain ranges characterized by linear or rectangular geometries. We employ Prisma to calculate the topography correction for the Bouguer anomaly by representing the DEM cells with prisms. This routine can further be used to determine the cross-sectional geometry of the flanks of the bedrock beneath the surface, which is the scope of this paper. Prisma was also used to integrate the model and survey results into a GIS environment. Finally, Prisma serves to illustrate the first-order effects of a complex structure on a station. Please note that although we focus our work on reconstructing the cross-sectional and hence the 2D geometry of the target overdeepening, we calculated the 3D gravity effect of the overdeepening fill on our cross-section, because the gravity at a point is the consequence of the entire (and thus 3D) sedimentary architecture of the overdeepening fill in close proximity to our target cross-section.

We approximated the overdeepening fill with prisms, which are constrained using a-priori information that is based on the bedrock map underneath our target overdeepenings (see Reber and Schlunegger[4] for thickness maps). Please also note that other gravity toolboxes exist[6] that can be employed for various settings in Earth sciences. However, since Prisma is

tailored towards addressing specific questions that are related to overdeepenings[3] (i.e., estimation of gravity contrasts between the bedrock and the overdeepening fill, estimation of the maximum depth of overdeepenings etc.), we employed our own code. Please see the main text for further explanations.

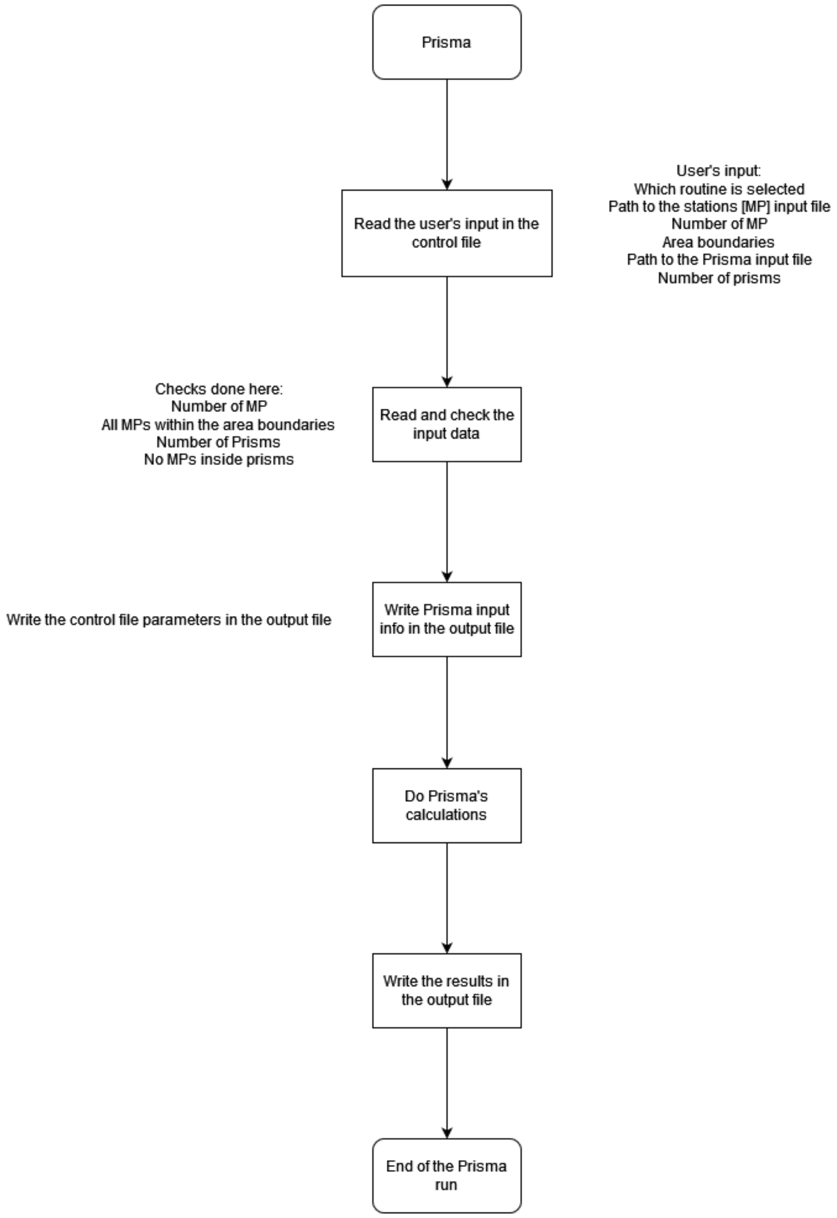

Figure S3: Simplified flowchart of Prisma showing the main steps done by the program.

S4: Estimation of density contrast between the bedrock and the overdeepening fill and maximum thickness of overdeepening fill using Prisma

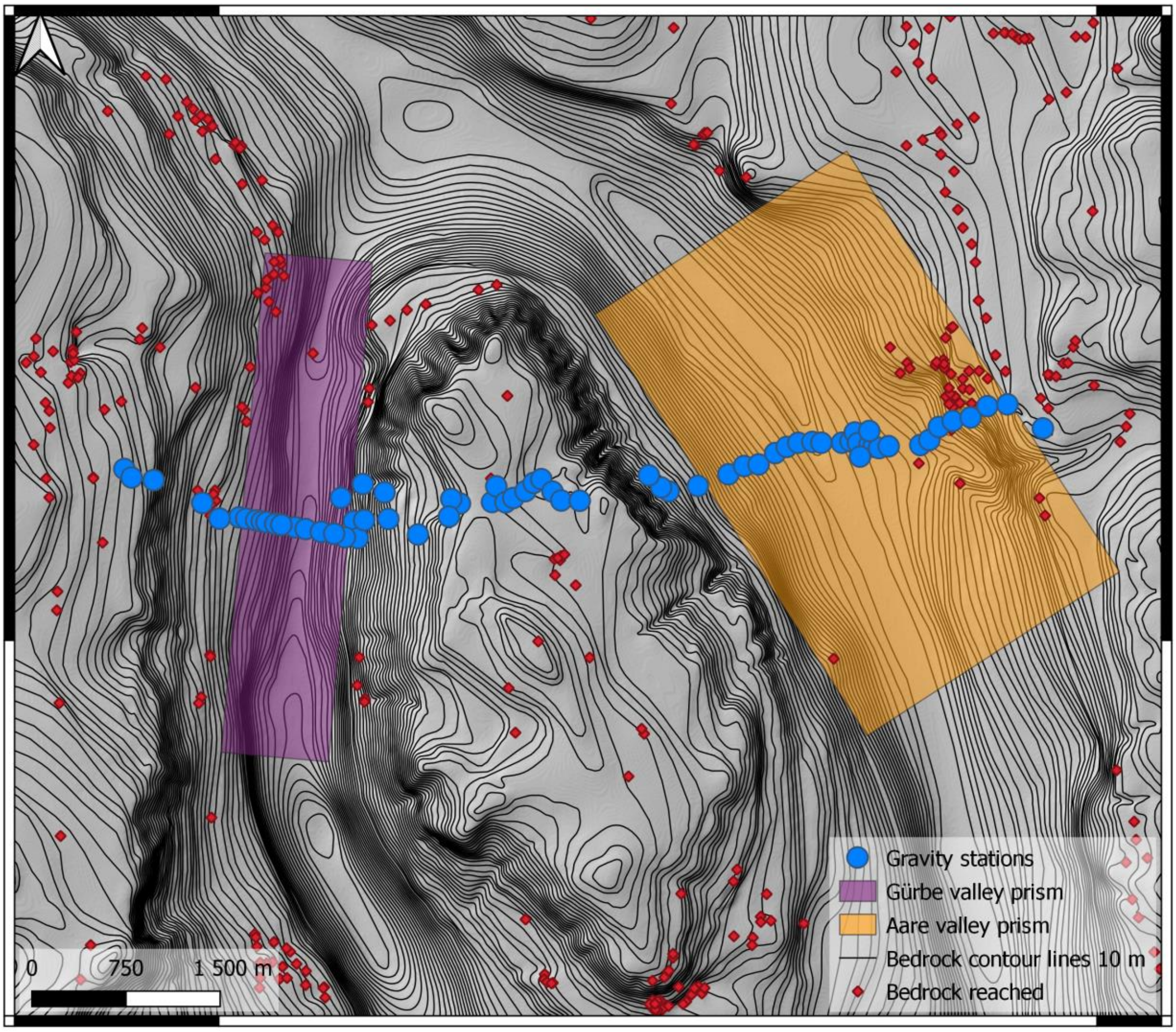

Figure S4a: Bedrock map[4], which was used as a basis to allocate the prisms' positions in the Gürbe and Aare valleys. Please note that the top surface of the Aare valley prism is located at an elevation of 520 m a.s.l., which corresponds to the station with the lowest elevation. Accordingly, for the Gürbe valley, we selected an elevation of 523 m a.s.l. The bedrock elevation model is openly accessible (SwissAlti3D, © swisstopo).

The estimation of the density of the Quaternary fill was accomplished indirectly[1] in the sense that the application of Prisma yields a density contrast between the bedrock and the sedimentary fill. For this purpose, for the Gürbe valley, we selected a prism with a width that fits both flanks and that runs parallel to the overdeepening as inferred by the bedrock topography map[4] (Figure S4a). The top of the prism is located at an elevation of 523 m. a.s.l., the lowest elevation close to our gravity section, and the centre of the prism is slightly to the north of it to better comply with the slightly curved geometry of the Gürbe valley. Likewise, the 2 km-long distance of the Prisma to the North and to the South from the centre, respectively, is defined by the geometry of the western flank of the Gürbe valley. The width of 860 m from the centre is constrained by the flank of the valley. The elevation profile (Figure S1) was positioned to be perpendicular to the prism and parallel

as much as possible to the gravity stations. The 5° clockwise rotation of the Swiss coordinate system axes, along the centre of the prism, was done to comply with the conditions that the model requires right-angled prisms that are oriented parallel to both coordinates' axes of the prism[7,8].

For the Aare valley, the prism was defined with its centre located at the same position as MP 3011, which corresponds to the valley centre. Both the NE and SW borders of the Aare valley prism were defined using the bedrock model from Reber and Schlunegger[4] as a criterion (Figure S4a). In particular, we allocated the borders along the margins where the upper part of the slope of the overdeepening ends (Figure S4a). The prism has a length of 2000 m in both directions and a total width of 2400 m. The elevation line is shifted to the North of the prism centre as it is the best compromise to fit the surface topography, which shows that the western flank of the Belpberg has a concave geometry. This profile also includes the stations at the centre of the valley. The prism is also offset to this line to best fit the information from the bedrock topography model of Reber and Schlunegger[4]. A counterclockwise rotation of 33° was operated onto the Swiss coordinate system axes for the same reasons as the Gürbe valley.

The results show that for the Gürbe valley, a maximum residual anomaly of -2.9 mGal (see Figure 4 main text) is reached for a density contrast between 400 and 500 kg/m^3, which correspond to densities of 2100 and 2000 g/cm^3 for the Quaternary sediments (Figure S4b). All other scenarios are associated with thicknesses and densities for the Quaternary material that are beyond what has been estimated so far[5]. A shortening of the prism by 100 m in each directions reduces the calculated residual anomalies by values that are smaller than our uncertainty, which is +/-0.13 mGal. Alternatively, an increase in the prism length by 200 m in each direction increases the signal at the station by less than the uncertainty. This then implies that for the Gürbe valley, the selected length of the prism and thus of our model is likely appropriate. For the Aare valley, for a maximum anomaly of -4.1 mGal, this approach yields a thickness of 210 m for the overdeepening fill, and density contrast of 500 kg/m^3 (Figure S4c). We note, however, that this thickness must be considered as a lower bound because available drilling data (see Figure 2c main text) imply a thicker Quaternary suite. Moreover, because it is a simplistic single prism model, the gravity effect on the flanks is overestimated, as they reach deeper than what

drillings have disclosed, which further biases the estimation of the thickness of the Quaternary fill.

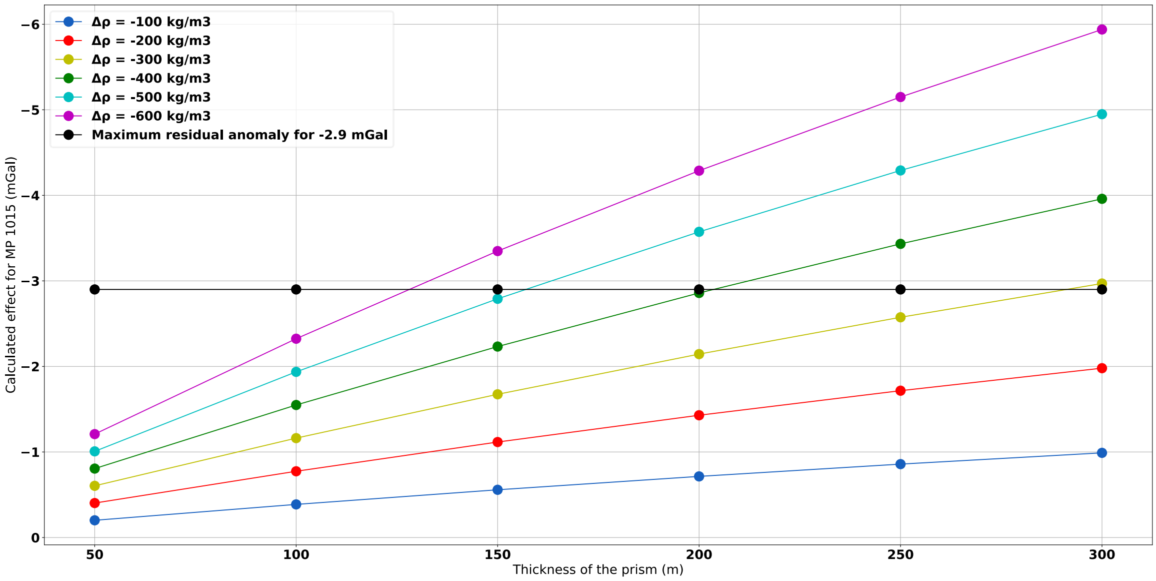

Figure S4b: Plot of the Prisma model results that are used to determine the maximum Quaternary sediment thickness and the density contrast between the Quaternary sediments and the bedrock in the Gürbe valley, following Kissling and Schwendener[3].

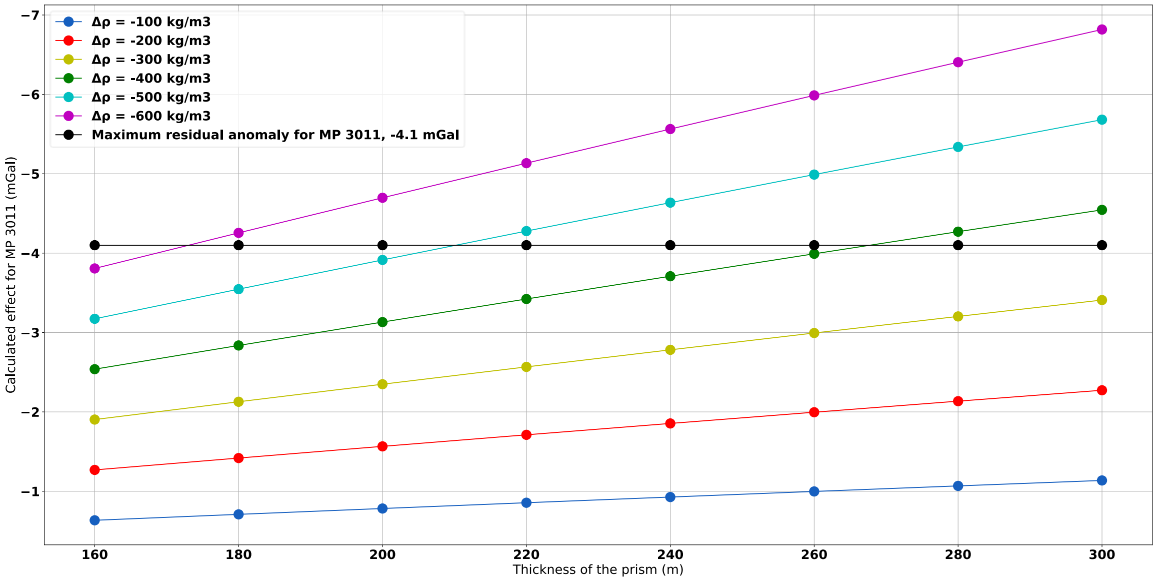

Figure S4c: Plot of the Prisma model results that are used to determine the maximum Quaternary sediment thickness and the density contrast between the Quaternary sediments and the bedrock in the Aare valley, following Kissling and Schwendener[3].

S5: Modelling of the residual gravity anomaly across the Gürbe valley using Prisma

The figures S5 present the model results of the residual gravity anomalies across the Gürbe valley. We started with a single prism (Figure S5a), and the first model (Figure S5b) represents an approximation of the overdeepening fill, which can be described, in

the simplest case, by one prism. The middle plot (Figure S5c) shows the modelling results where a V-shaped geometry was applied to the valley, whereas the modelling results of the following plots (Figure S5d, S5e and S5f) are based on the inference of an asymmetric U shape geometry for the overdeepening. For Figures S5b to S5f, the blue and green dots represent the observed gravity at the stations (see map above for legend), whereas the orange dots represent the modelled residual anomaly for each station. The black bars on each dot represent the uncertainty of +/- 0.13 mGal. The black dashed lines indicate the valley width. The maximum residual anomaly value of -2.9 mGal is indicated by the red dashed line on Figure S5b. The figures additionally show the prism(s) in black, the elevation profile as a blue line with the blue dots indicating the stations' positions. The red dashed line indicates the bedrock model profile from Reber and Schlunegger[4], with the diamonds indicating bedrock depths acquired through drillings.

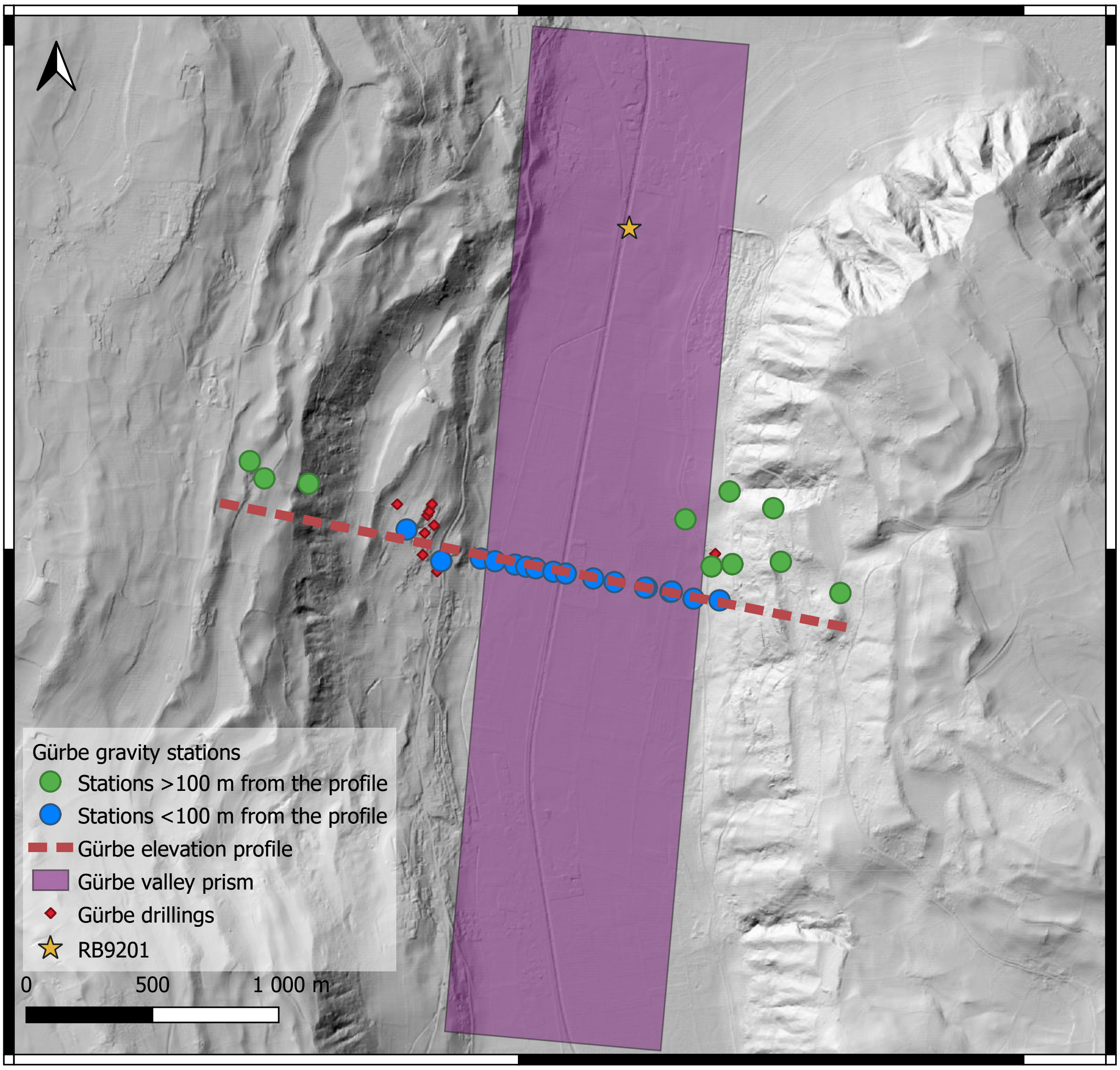

Figure S5a: Map of the Gürbe valley prism. The prism is in purple, the blue dots show the stations less than 100 m away from the profile, represented as a red dashed line, while the green dots show the stations over 100 meters away from the profile. The red diamonds represent the drillings used to constrain the depth for the models and the star

indicates the drilling used for constraining the maximum depth and for the sedimentary log (Figure 6 see main text). The DEM is openly accessible (SwissAlti3D, © swisstopo).

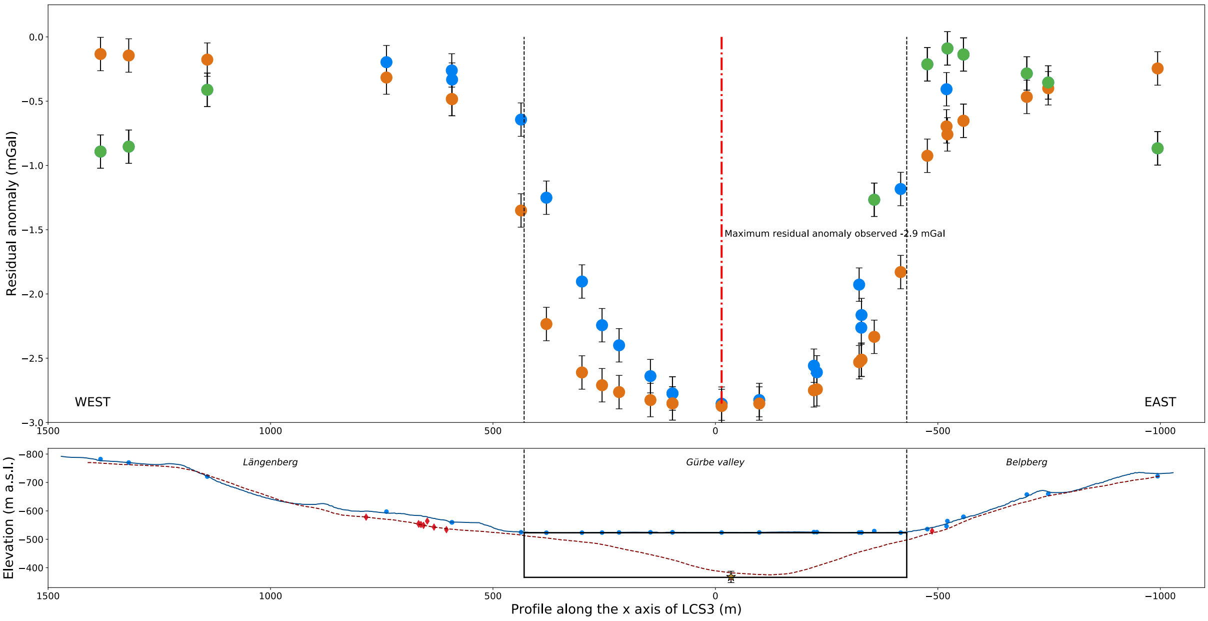

Figure S5b: Modelling results where the overdeepening fill is approximated by one single prism. There is a large misfit between observed and modelled residual Bouguer anomalies, indicating that we considered too much Quaternary material on the overdeepening's flank, as the half width of the anomaly does not fit with a too high amplitude. The maximum anomaly, however, is properly modelled even for the stations where the anomaly is 90% of the maximum, indicating a likely wide geometry at depth for the overdeepening. The blue and green dots represent the observed gravity at the stations (see map above for legend) while the orange dots represent the modelled residual anomaly for each station. The blue line and blue dots show the elevation profile with the elevation of the stations, and the red dashed line and red diamonds indicate the bedrock model profile[1] and the bedrock depth from drillings, respectively.

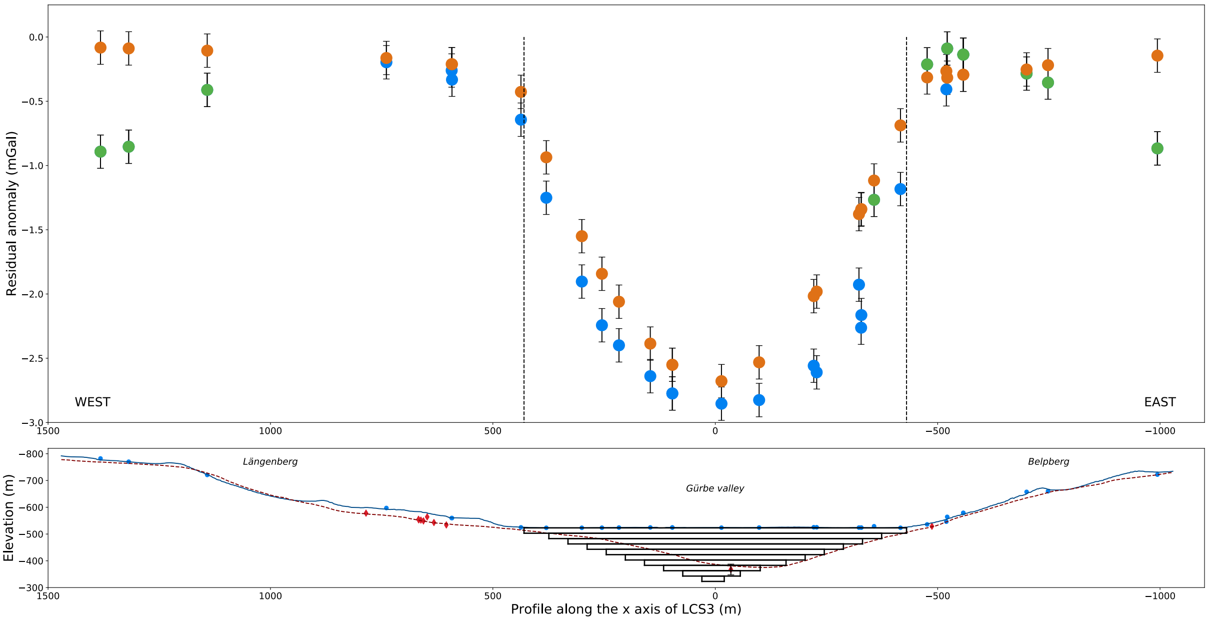

Figure S5c: Modelling results where the overdeepening fill is approximated by a V-shaped geometry. We see that such a solution results in an underestimation of the residual anomalies particularly on the lateral flanks with a lower amplitude in the anomaly than observed. Moreover, for the stations with a gravity effect within 90% of the maximum anomaly the calculated values are underestimating the observations, and the maximum anomaly is reached only for a deeper maximum depth than indicated by the drilling. (See figure S5b for the legend).

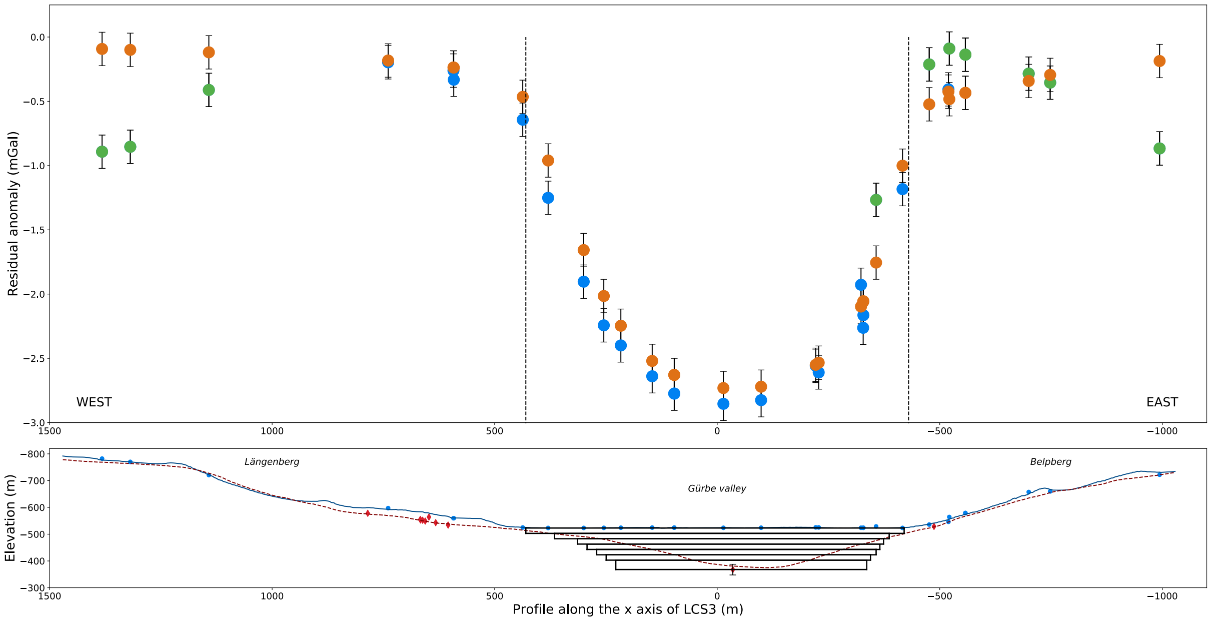

Figure S5d: We iteratively increased the lateral extents of the prisms, thereby considering the case of a U-shaped overdeepening geometry with an asymmetry, where the western flank is less steep than the eastern flank. The results show that the fit on both sides is still not good enough in relation to the proposed uncertainty in the gravity measurements. We do see that the amplitude of the residual gravity is generally matched and that the difference between calculated and observed values is quite low. The input and output of this model is available in the supplementary dataset Prisma, and it is used as an example to run the code, labelled example1_ushape. (See figure S5b for the legend)

S6: Modelling of the residual gravity anomaly across the Aare valley using Prisma

The figures in this section show the results of the models of the Aare valley residual anomaly. The starting process follows the same logic as for the Gürbe situation. In particular, we started with a single prism (Figure S6a) in the middle of the valley to model the overdeepening fill. However, in contrast to the Gürbe valley, a single prism does not give a satisfactory first approximation of the residual anomaly as the model anomaly has a higher amplitude than the overall observations (Figure S6b). For this reason, we used two prisms. A wider top prism is used to approximate the shallower part of the overdeepening, whereas a narrower but much thicker prism is assigned for the depth of the overdeepening (Figure S6c). We then tested the cases where we considered both V- and U-shaped geometries (Figures S6d and S6e). The observed data measured at the stations are indicated with blue dots, while the modelled values are marked with orange dots. Both values have an uncertainty of +/- 0.13 mGal indicated with black bars. Black dashed lines indicate the valley width, used to define the top prism width. The bottom plots indicate the elevation profile with a blue line and the stations elevation with blue dots. The drillings that reached the bedrock and that are used as constraints are marked in red, with the two yellow and white stars indicating two drillings that did not reach the bedrock but that are still used for constraining (Figure 3 main text) the geometry.

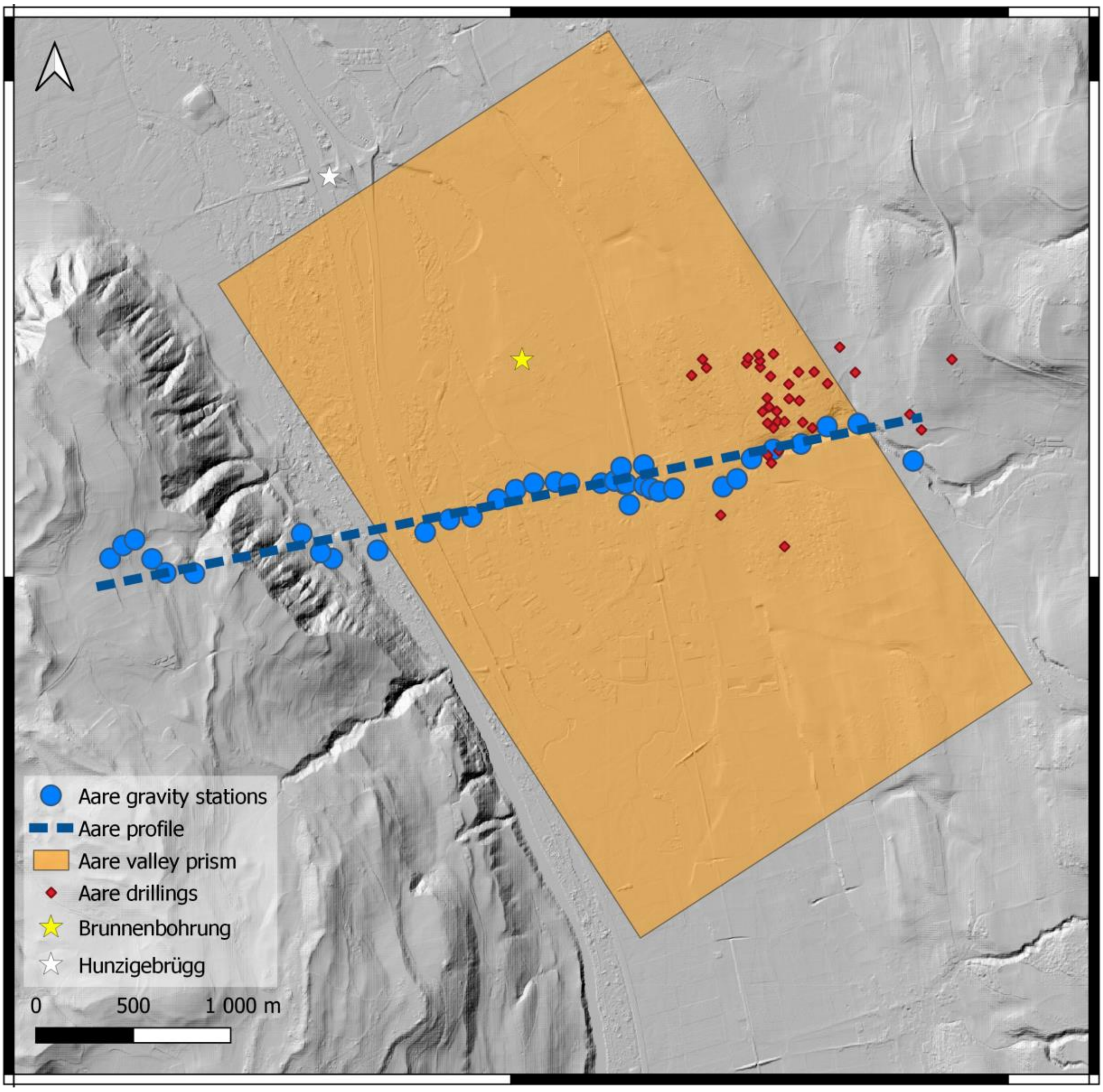

Figure S6a: Map of the Aare valley prism. The prism is in orange, the blue dots show the stations. Because of the nonlinear organization of the stations, we do not distinguish between stations that are closer or farther away from the gravity profile. The red diamonds represent the drillings used to constrain the depth for the models. The two stars indicate drillings that provided a minimum depth constraint, and they are used for the sedimentary log of this paper (Figure 6 see main text). The DEM is openly accessible (SwissAlti3D, © swisstopo).

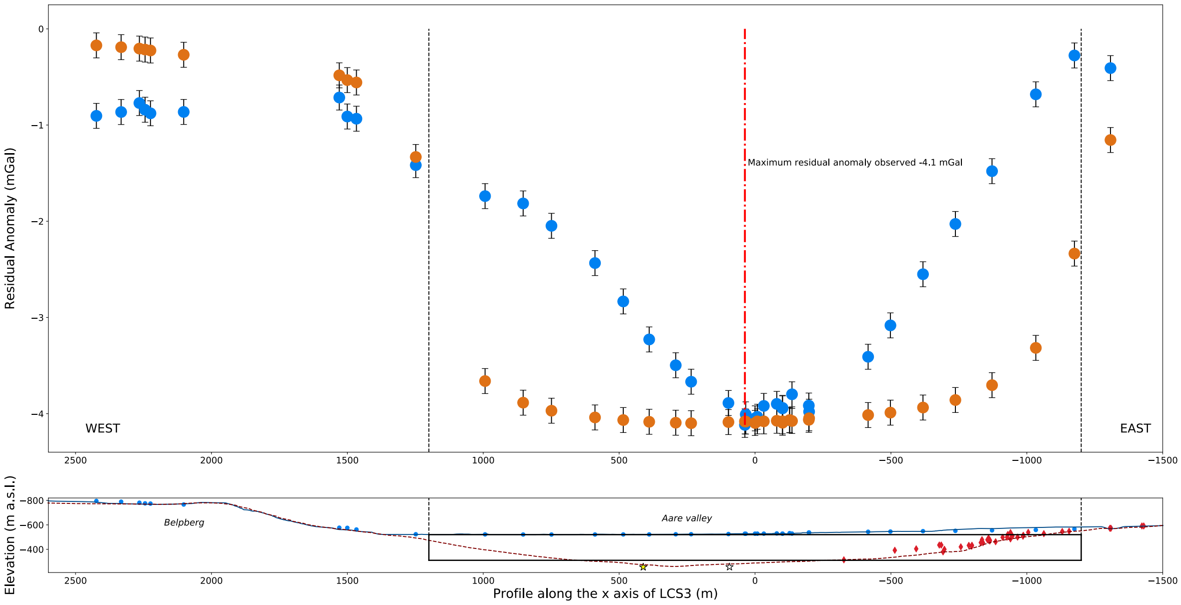

Figure S6b: Result of the first model of the Aare. The red dashed line shows the maximum anomaly of -4.1 mGal. The single prism does not reach all the way to the western border of the valley (Belpberg), yet the effect is much higher than observed. Also, whereas the maximum anomaly of -4.1 mGal is reached, the amplitude is larger than observed and the results do not fit the observations on the flanks of the overdeepening. This indicates that the overdeepening fill needs to be modelled with two prisms, a narrower prism at depth and a wider prism close to the surface. The blue dots are the observed residual anomaly values at the stations, the orange ones are the modelled residual anomalies. The black bars represent the uncertainty of +/- 0.13 mGal. The black dashed line indicates the width of the valley and subsequently of the prism. The blue line is the elevation profile with the blue dots showing the stations. The red diamonds are the drillings that reached the bedrock and that are used as constraints. The two stars (yellow and white) are drillings (see Figure 2b and 6 in main text) used as constraints, yet they did not reach the bedrock. The red dashed line is the profile of the bedrock model from[4].

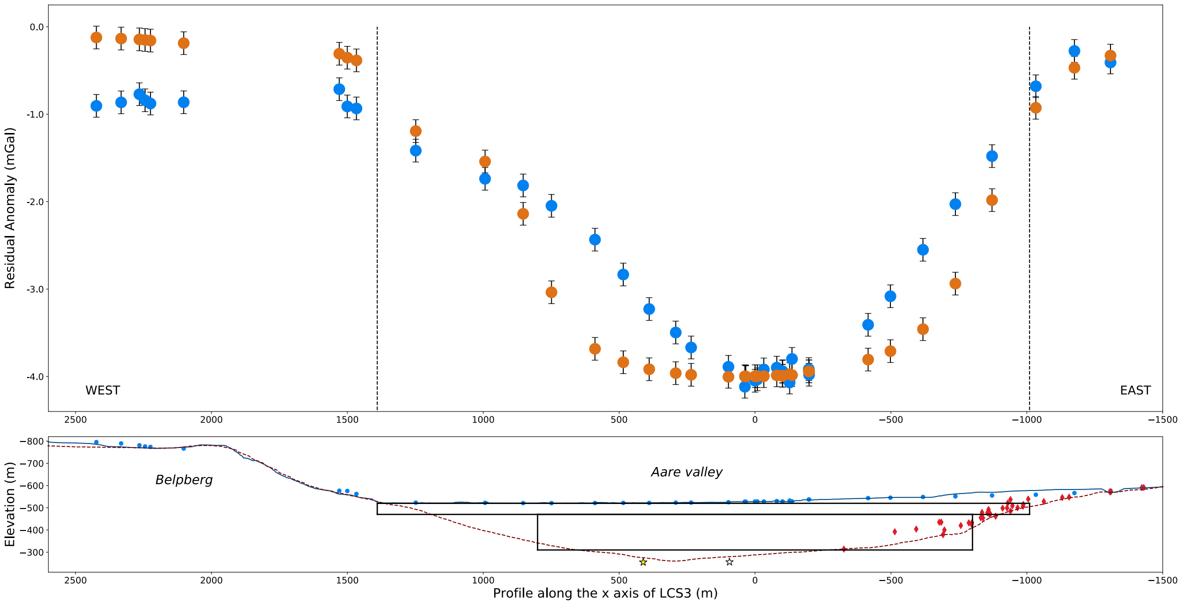

Figure S6c: Results of the Aare valley modelled with two prisms (see Figure S6b for the legend). The results for this second model are much better, but the amplitudes for the maximum anomaly and the half-width are still too large. Yet it is much closer than the solution illustrated in Figure S6b (above). The flanks of the overdeepening have now been properly approximated, confirming the presence of a much narrower overdeepening geometry at depth than at the surface. (See Figure S6b for the legend).

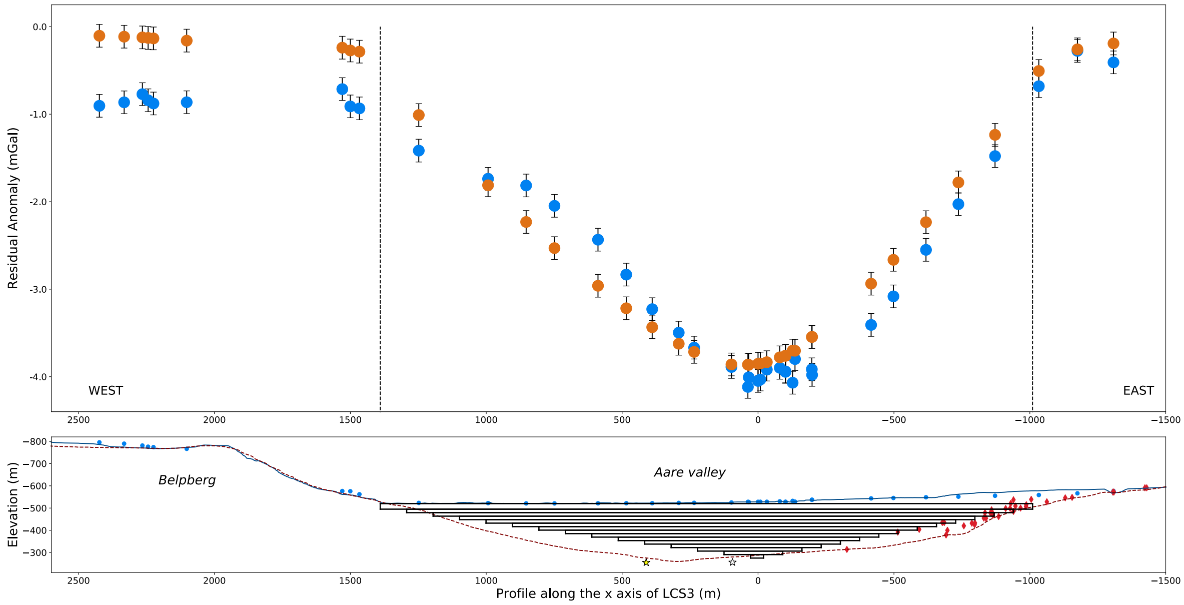

Figure S6d: Results of the V-shaped geometry model of the Aare overdeepening. The results show a fit of the general amplitude of the residual anomaly, however the fit around the maximum anomaly is not good, as the model results appear to underestimate the observed effect. The residual anomaly is slightly underestimated in this model. In the same sense, the anomaly on the western border is not properly modelled with this symmetric geometry. This model indicates that the slope of the Aare overdeepening is not as steep as the Gürbe valley overdeepening. It also indicates the need for an asymmetric geometry to approximate the anomaly pattern on the western flank. The input and output of this model is available in the supplementary dataset Prisma, and it is used as an example to run the code, labelled example2_vshape. (See Figure S6b for the legend).

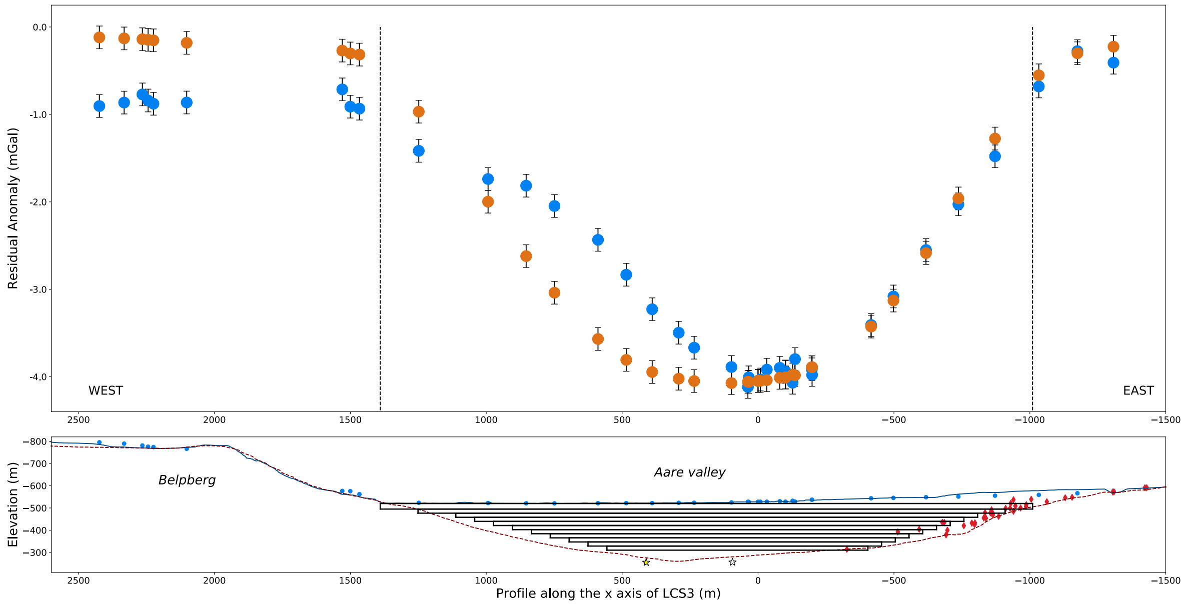

Figure S6e: Results of the U-shaped geometry model of the Aare overdeepening. Like above, the results here show a good fit of the general amplitude of the residual anomaly. In particular, the fit is reasonably good around the maximum of the anomaly, but it is worse on the western flank where the modelled amplitudes are too high. The effect on the eastern flank is slightly overestimated. This model suggests that a wide and flat geometry at the bottom of the overdeepening is very likely, and that the geometry of the eastern flank lies in between this one and the solution illustrated in Figure S6d (see above). It also shows the need for an asymmetric western flank, with an extended plateau at depth. (See Figure S6b for the legend).

Please note we are not capable of accounting for a possible depth dependency of the gravity values at least for the Holocene mud. However, the consideration of a lower density for the Holocene material and a higher density for the pre-LGM deposits will not alter our conclusion that the upper part of the target overdeepenings is much wider than

the lower one (see Figures S6).

S7: Application of a different density contrast to the Aare overdeepening.

Figure S4c suggest that a density contrast of 450 kg/m^3 could also be suitable to explain the maximum residual anomaly measured across the Aare valley. Using such a density contrast, however, does not yield a geometry that fits the both the gravity and the depth contrast offered by drillings on the eastern flank (Figure S7). These results support our choice of the same density contrast for both valleys.

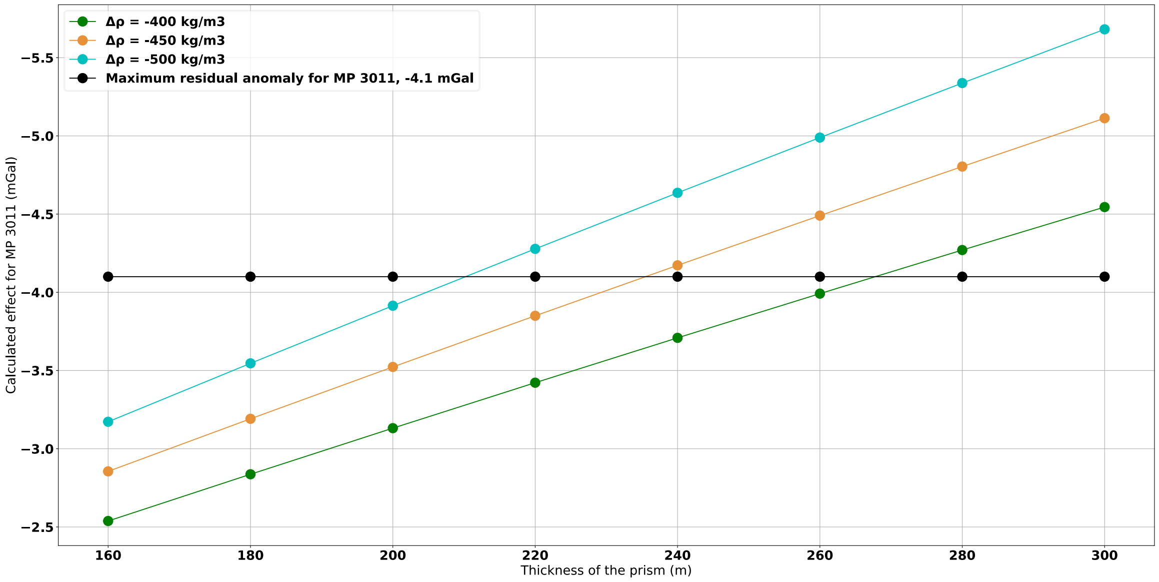

Figure S7a: Similar to Figure S4c, this plot indicates the density contrasts and prism thickness needed to fit the maximum anomaly of the Aare overdeepening. Here we show that a contrast of 450 kg/m3 could be a valid value to be used, because the prism thickness to explain the maximum residual anomaly would be similar to the proposed value in the bedrock model[4].

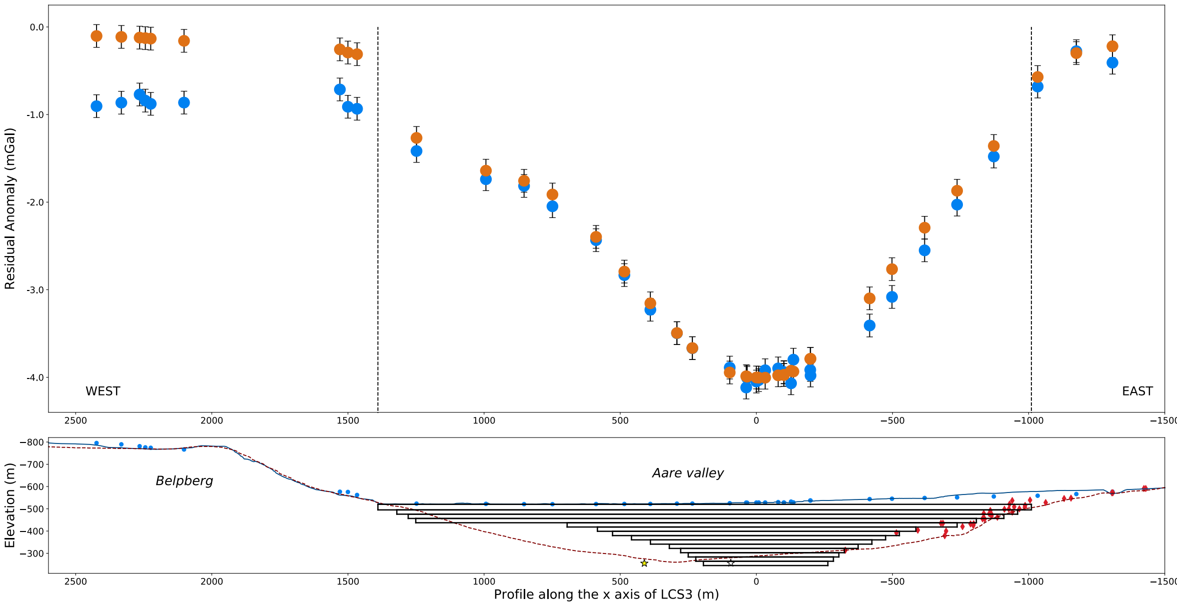

Figure S7b: Plot of the model that best fits the geometry of the Aare overdeepening on its eastern flank. This model is based on a density contrast of 450 kg/m3 as suggested by Figure S7a. Nevertheless, there is a misfit with the residual anomalies. In particular, this particular geometry does not provide an overdeepening volume that is large enough to match the observed gravity. Please see Figure S6 for the legend.

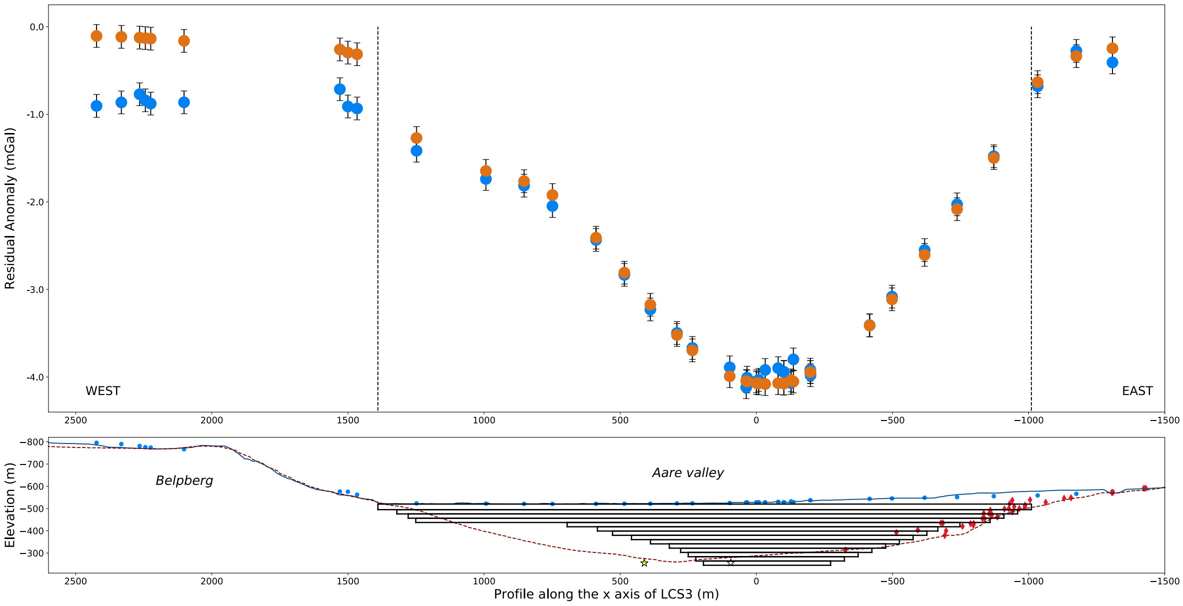

Figure S7c: Plot of the model that best fits the residual anomaly of the Aare overdeepening on its eastern flank. This model is based on a density contrast of 450 kg/m3 as suggested by Figure S7a. Nevertheless, we observe that there is a misfit with the constrains offered by the drillings. The overdeepening width required to successfully model the residual anomalies exceeds the constrains given by the drillings. Please see Figure S6 for the legend.

References

[1]Beck, P. & Rutsch, P. Geologische Karte der Schweiz, Kartenblatt Münsingen-Konolfingen-Gerzensee-Heimberg. Schweiz. Geol. Komm. (1949).

[2]Spicher, A. Geologische Karte der Schweiz 1:500'000. Schweiz. Geol. Komm. (1972).

[3]Kissling, E. & Schwendener, H. The Quaternary sedimentary fill of some Alpine valleys by gravity modeling. Eclogae geol. Helv., 83, 311-321 (1990).

[4]Reber, R. & Schlunegger, F. Unravelling the moisture sources of the Alpineglaciers using tunnel valleys as constraints. Terra Nova, 28, 202-211 (2016).

[5]Schwenk, M. et al. From glacial erosion to basin overfill: A 240 m-thick overdeepening-fill sequence in Bern, Switzerland. Sci. Drilling, in press (2021).

[6]Schmidt, S., Anikiev, D., Götze, H. J., Gomez Garcia, À., Gomez Dacal, M. L., Meeßen, C., Plonka, C., Rodriguez Piceda, C., Spooner, C., & Scheck-Wenderoth, M. IGMAS+-a tool for interdisciplinary 3D potential field modelling of complex geological structures. In *EGU General Assembly Conference Abstracts* (p. 8383) (2020, May).

[7]Nagy, D. The gravitational attraction of a right rectangular prism. Geophyscis, 31, 362-271 (1966).

[8]Banerjee, B. & DasGupta, S.P. Gravitational attraction of a rectancular parallelepiped. Geophysics, 42, 1053-1055 (1977).
